# Graphene Quantum Dot Sensitized Heterojunctions Induce Tumor‐Specific Cuproptosis to Boost Sonodynamic and Chemodynamic Enhanced Cancer Immunotherapy

**DOI:** 10.1002/advs.202410606

**Published:** 2024-12-24

**Authors:** Lang Yan, Liang Chang, Yijun Tian, Jinyan Hu, Zhi Cao, Xiang Guo, Bijiang Geng

**Affiliations:** ^1^ Department of Health Toxicology Faculty of Naval Medicine Naval Medical University Shanghai 200433 China; ^2^ Department of Emergency and Critical Care Shanghai Changzheng Hospital Second Affiliated Hospital Naval Medical University Shanghai 200003 China; ^3^ School of Environmental and Chemical Engineering Shanghai University Shanghai 200444 China; ^4^ Department of Urology Changhai Hospital Naval Medical University Shanghai 200433 China; ^5^ Department of Orthopaedics Shanghai Changzheng Hospital Second Affiliated Hospital Naval Medical University Shanghai 200003 China

**Keywords:** Cu_2_O nanocubes, graphene quantum dots, immunotherapy, sonodynamic therapy, tumor‐specific cuproptosis

## Abstract

Cuproptosis that utilizes copper ionophore to induce programmed cell death holds promise for enhancing the effectiveness of conventional anticancer therapies and triggering efficient adaptive immune responses. However, the non‐tumor‐specific release of Cu ions can induce cuproptosis and cause irreversible damage to normal tissues. To maximize the therapeutic effects of tumor‐specific cuproptosis, this work reports for the first time the regulation of degradation behaviors of Cu‐based nanomaterials using graphene quantum dots (GQDs) as a protection layer. The deposition of GQDs not only avoids the degradation of Cu_2_O nanocubes under normal physiological conditions, but also sensitizes their sonodynamic activity due to the formation of Z‐scheme heterojunctions. The tumor‐specific released Cu ions achieve the cascade amplification of reactive oxygen species (ROS) generation through Cu^+^‐mediated Fenton‐like reaction and Cu^2+^‐facilitated GSH depletion. More importantly, the immunosuppressive tumor microenvironment (TME) can be reversed by the greatly enhanced ROS levels and high‐efficiency cuproptosis, ultimately inducing immunogenic cell death that promotes robust systemic immune responses for the eradication of primary tumors and suppression of distant tumors. This work provides a novel paradigm for the integration of SDT, CDT, cuproptosis, and immunotherapy in a controlled manner to achieve tumor‐specific antitumor therapy by controlling the degradation behaviors of Cu‐based nanomaterials.

## Graphical abstract

Graphene quantum dots are utilized as a protection layer to regulate the degradation behaviors of Cu‐based nanomaterials. The tumor‐specific released Cu ions achieve the cascade amplification of ROS generation. The immunosuppressive tumor microenvironment can be reversed by the greatly enhanced ROS levels and high‐efficiency cuproptosis, ultimately inducing robust systemic immune responses for the eradication of primary tumors and suppression of distant tumors.

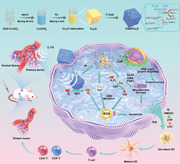

## 1 Introduction

Metal ions are indispensable for an organism's normal functions, playing crucial roles in diverse physiological processes.^[^ ^1^, ^2^, ^3^, ^4^, ^5^ ^]^ While biosystems usually maintain copper concentration through evolutionarily conserved homeostatic mechanisms, exceeding a certain threshold can lead to severe toxicity and result in cell death.^[^ ^6^, ^7^ ^]^ Copper ions effectively inhibit cancer cell proliferation by inducing biocatalysis, altering metabolism, disrupting signal transduction, and causing DNA damage, thereby avoiding susceptibility to drug resistance.^[^ ^8^, ^9^, ^10^ ^]^ For example, the interaction between Cu^+^ and lipoylated components within the tricarboxylic acid (TCA) cycle could trigger the aggregation of lipoylated proteins and lead to the degradation of iron‐sulfur (Fe‐S) cluster proteins, ultimately resulting in cell death through cuproptosis.^[^ ^11^, ^12^, ^13^ ^]^ The mechanism of cuproptosis should be distinguished from the traditional pathways of cell death, such as apoptosis, pyroptosis, and ferroptosis.^[^ ^14^, ^15^, ^16^ ^]^

While examining the mechanism of cuproptosis and the unique characteristics of the tumor microenvironment (TME), cuproptosis‐based treatments continue to face the following challenges that require resolution. First, the currently developed cuproptosis inducers are mainly limited to copper ionophores, such as elesclomol, which transported Cu^+^ into tumor cells and induce cuproptosis.^[^ ^17^, ^18^ ^]^ Nevertheless, it remains a challenge to achieve cuproptosis in tumor cells owing to the fluctuations in Cu^+^ metabolism and the limited tumor accumulation of copper ionophores.^[^ ^19^, ^20^ ^]^ More importantly, the systemically distributed copper ionophores after intravenous administration would also induce cuproptosis in normal tissues, causing irreversible damage to normal tissues.^[^ ^21^ ^]^ Thus, it is crucial to rationally design TME‐responsive nanoplatforms functioning as copper ionophores to realize tumor‐specific cuproptosis. Second, the interaction between copper and the highly expressed glutathione (GSH) in TME can hinder the copper's binding to lipoylated components in the TCA, potentially diminishing the efficacy of cuproptosis.^[^ ^22^, ^23^, ^24^ ^]^ Therefore, on the basis of achieving tumor‐specific cuproptosis, realizing the consumption of GSH is the second challenge that must be overcome to further enhance cuproptosis effects. Third, in order to achieve simultaneous induction of cuproptosis and depletion of GSH, various Cu‐based nanomaterials containing valence Cu^+^/Cu^2+^ have been reported for TME‐regulating‐enhanced cuproptosis and chemodynamic therapy (CDT).^[^ ^18^, ^25^, ^26^, ^27^ ^]^ However, the degradation characteristics of Cu‐based nanomaterials are difficult to control, in which the non‐specific degradation could lead to acute toxicity in normal tissues, and the non‐degradation features could restrain the release of Cu ions, greatly reducing the effectiveness of cuproptosis and CDT.^[^ ^28^, ^29^, ^30^ ^]^ Overall, the rational regulation of degradation rate of Cu‐based nanomaterials could not only prevent undesirable release of Cu ions in normal tissues, but also possess tumor‐specific degradation characteristics to maximize the therapeutic effects of cuproptosis.

Cuproptosis also represents a type of copper‐dependent immunogenic cell death (ICD) that triggers immune response by releasing damage‐associated molecular patterns (DAMPs).^[^ ^30^, ^31^, ^32^ ^]^ On this basis, the immunosuppressive TME could be reversed by the induction of cuproptosis, subsequently achieving the cuproptosis‐enhanced immunotherapy. In another respect, the ICD effects could also be triggered by ROS‐mediated tumor therapy, such as photodynamic therapy (PDT) and sonodynamic therapy (SDT).^[^ ^33^, ^34^, ^35^, ^36^, ^37^ ^]^ Among them, SDT can overcome the inherent defects of PDT owing to the higher tissue penetration depth (>10 cm), exhibiting the advantage of treating tumors that are not limited by the depth of the tumor location.^[^ ^38^, ^39^, ^40^, ^41^ ^]^ However, SDT‐induced ICD effects are substantially restricted by the insufficient ROS yield owing to the low‐efficiency sonosensitizers and complex TME.^[^ ^42^, ^43^ ^]^ The poor aqueous stability, potential phototoxicity, instable chemical property of traditional organic sonosensitizers and the wide bandgap as well as fast electron‐hole pair recombination of inorganic nanomaterials would result in the low ROS yield.^[^ ^44^, ^45^, ^46^ ^]^ Recently, the fabrication of heterojunctions with matched bandgap has been explored to suppress the recombination of electron‐hole pairs.^[^ ^47^, ^48^, ^49^ ^]^ Based on this situation, developing heterojunction sonosensitizers based on Cu‐based nanomaterials is expected to control the degradation characteristics of cuproptosis inducers, which could not only achieve cascade amplification of ROS yield through SDT and TME regulation, but also further amplify the ICD effects through tumor‐specific cuproptosis, ultimately achieving a “1 + 1> 2” therapeutic effects.

**Scheme 1:**
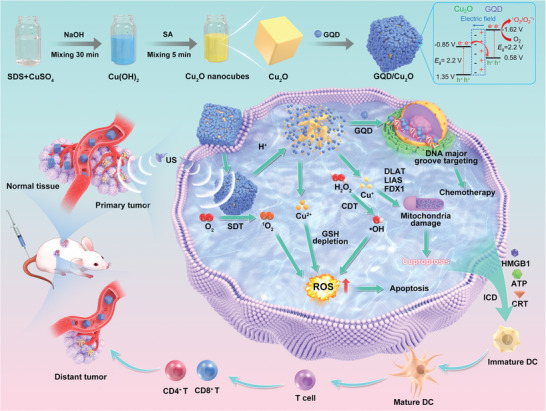
Diagram depicting the formation of GQD/Cu_2_O heterojunctions to boost tumor‐specific cuproptosis for augmented SDT/CDT/immunotherapy.

As a semiconductor oxide with exceptional features, Cu_2_O could serve as an ideal sonosensitizer and nanozyme owing to the narrow bandgap and Cu^+^‐mediated Fenton‐like reaction.^[^ ^29^, ^50^ ^]^ It is well known that the catalytic efficiency of Cu^+^ surpasses the widely reported Fe‐based CDT agents by 160‐fold.^[^ ^51^, ^52^, ^53^ ^]^ More importantly, due to the degradation and easy oxidation characteristics, Cu_2_O can be used as a potential copper ionophore for Cu^+^‐mediated cuproptosis. However, the degradation and oxidation of Cu_2_O also occur under normal physiological conditions, leading to cuproptosis and Fenton‐like reaction in normal cells, which would cause irreversible damage to normal tissues.^[^ ^16^, ^54^ ^]^ Herein, we utilized graphene quantum dot (GQD) topoisomerases inhibitors as a protection layer to control the degradation behaviors of Cu_2_O for the realization of tumor‐specific cuproptosis and CDT. The deposition of GQDs not only prevented the degradation of Cu_2_O nanocubes under normal physiological conditions to avoid the side effects on normal cells, but also enhanced the sonodynamic activity of Cu_2_O sonosensitizers owing to the formation of Z‐scheme heterojunctions, which could inhibit the recombination of electron‐hole pairs. The pH‐responsive degraded GQD/Cu_2_O heterojunctions would release GQDs, Cu^+^, and Cu^2+^ in the acidic TME, which could achieve the cascade amplification of ROS production owing to the Cu^+^‐mediated Fenton‐like reaction and Cu^2+^‐facilitated GSH depletion. More importantly, the released Cu^+^ realized the tumor‐specific cuproptosis through causing DLAT oligomerization and mitochondrial dysfunction. Notably, GQD/Cu_2_O heterojunctions‐mediated SDT, CDT, and cuproptosis induced severe cellular damage to release abundant DAMPs for the activation of ICD effects, which further reversing the immunosuppressive TME and inducing robust systemic immune responses. Moreover, the tumor‐specific released GQDs could target the major groove of DNA, inhibit topoisomerase I/II, and cause DNA damage to induce apoptosis for the enhanced chemotherapy based on our previous report.^[^ ^55^ ^]^ Owing to these favorable features, the satisfactory therapeutic effects of primary and distant tumors were achieved through the tumor‐specific cuproptosis‐enhanced SDT, CDT, and immunotherapy (**Scheme** **1**).

## 2 Results and Discussion

**Figure 1:**
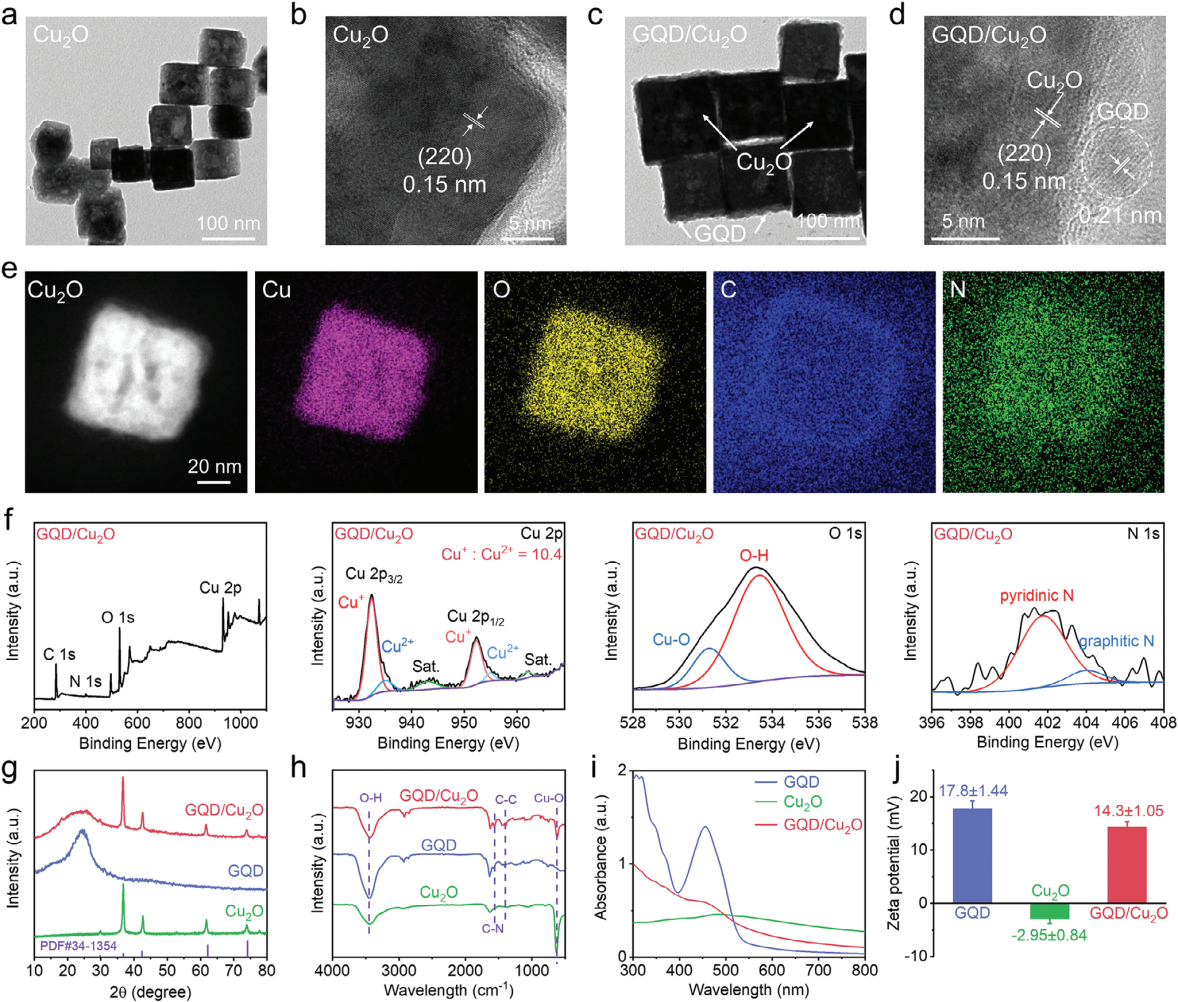
a–d) TEM and HRTEM images of Cu_2_O and GQD/Cu_2_O. e) TEM element mapping of GQD/Cu_2_O heterojunction. f) XPS spectra of GQD/Cu_2_O. g–j) XRD pattern g), FTIR h), absorption spectra i), and Zeta potential j) of GQD, Cu_2_O, and GQD/Cu_2_O. Data are presented as the mean ± SD. (*n* = 3).

TEM image in **Figure** **1**a showcased the cubic structure of Cu_2_O, indicating that the edge lengths of Cu_2_O nanocubes were ≈86 nm. DLS was employed to ascertain the hydrodynamic size of Cu_2_O nanocubes (92 nm) (Figure S1, Supporting Information). The good crystallinity of Cu_2_O nanocubes was confirmed by the HRTEM image (Figure 1b), exhibiting the (220) plane with lattice spacing of 0.15 nm. Additionally, XRD pattern demonstrated the high crystallinity of Cu_2_O nanocubes, displaying four distinct characteristic peaks (Figure 1g). These results agreed with the standard Cu_2_O structure (PDF#34‐1354). The successful synthesis of Cu_2_O nanocubes was also verified by FTIR, which exhibited the stretching vibration peak of Cu‐O (Figure 1h). Moreover, XPS was utilized to uncover the rationale behind the easy oxidability of Cu_2_O. Cu and O elements were evident in the XPS spectrum of Cu_2_O (Figure S2, Supporting Information). By analyzing the high‐resolution Cu 2p spectrum, it was determined that Cu_2_O nanocubes contain both Cu^+^ and Cu^2+^ valence states. The emergence of Cu^2+^ in Cu_2_O could be ascribed to the partial oxidation of Cu_2_O owing to their unstable properties. Moreover, peaks attributed to Cu‐O and O‐H were observed in the high‐resolution O 1s spectrum. The Cu_2_O nanocubes demonstrated a negative charge due to the hydroxy functional group present, as shown by the Zeta potential measurements (Figure 1j).

Given the instability of Cu_2_O, we chose to employ GQDs with exceptional water solubility as a protective barrier on the Cu_2_O nanocubes. GQDs, modified with N‐heterocycles, were synthesized through a simple microwave reaction using julolidine as a containing‐N precursor. XPS analysis presented in Figure S3 (Supporting Information) exhibited the presence of pyridinic N, which would be protonated in an aqueous solution and thereby lead to a positive charge characteristic of GQDs. The positive charge features of GQDs were then demonstrated by the Zeta potential (Figure 1j), which was determined to be 17.8 ± 1.44 mV at the low concentration (20 µg mL^−1^). By virtue of the strong positive charge characteristics, GQDs can target the DNA major groove of cancer cells and cause DNA damage by inhibiting the activity of topoisomerase I/II, which was demonstrated in our previous report.^[^ ^55^ ^]^ Considering that the phosphorylated H2AX (γ‐H2AX) was a marker of double‐strand breaks of DNA, we evaluated the expression levels of phosphorylated H2AX (γ‐H2AX) in 4T1 cells after different treatments. As shown in Figure S4 (Supporting Information), the expression levels of γ‐H2AX in 4T1 cells were significantly increased after treating GQD, demonstrating that the DNA damage was induced by GQD.

Due to the robust electrostatic interaction, the GQDs with a positive charge were readily attached to the surface of the Cu_2_O with a negative charge. TEM image (Figure 1c) was the first indication of successful heterojunction fabrication, revealing a thin layer of GQD on Cu_2_O. In addition, TEM image of GQD/Cu_2_O showed higher contrast, which also suggested the presence of thin GQD layer. We conducted HRTEM characterization to further confirm the loading of GQDs. The edge of GQD/Cu_2_O heterojunctions displayed a distinct lattice spacing of 0.21 nm, as shown in Figure 1d, which was attributed to the deposition of GQD. Furthermore, the element mapping of GQD/Cu_2_O heterojunctions also revealed the presence of C and N elements from GQDs (Figure 1e), demonstrating the successful fabrication of the GQD/Cu_2_O heterojunction. Furthermore, XRD pattern of heterojunctions displayed a broad diffraction peak at ≈25°, which was identified as GQDs, as seen in Figure 1g. C‐C and C‐N was both detected in the FTIR spectra of GQDs and GQD/Cu_2_O heterojunctions (Figure 1h). Peaks of C and N elements were detected in the survey spectrum of heterojunctions during the XPS analysis (Figure 1f). Pyridinic N was identified in the high‐resolution N 1s spectrum of GQD/Cu_2_O, confirming its presence in the pristine GQDs as well. The absorption peak at 450 nm, characteristic of GQDs, was also observed in GQD/Cu_2_O (Figure 1i), providing strong evidence for the successful formation of heterojunctions.

**Figure 2:**
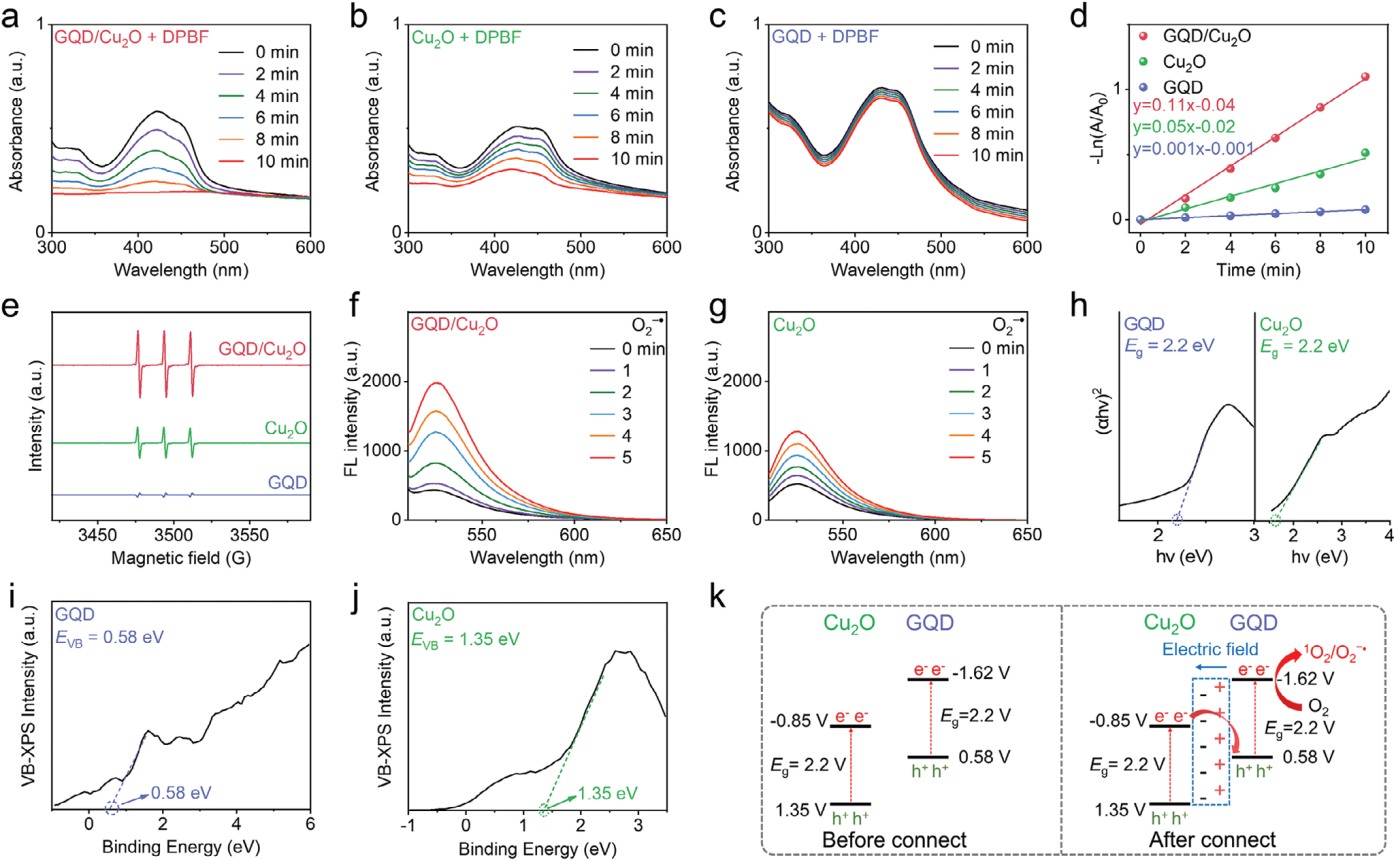
a–d) Measurements a–c) and comparison d) of the ^1^O_2_ generation of GQD, Cu_2_O, and GQD/Cu_2_O. e) ESR spectra of GQD, Cu_2_O, and GQD/Cu_2_O. f,g) Measurements of the O_2_ˉ^•^ generation of GQD, Cu_2_O, and GQD/Cu_2_O. h–j) Measurements of the bandgap and *E*_VB_ of GQD and Cu_2_O. k) Within GQD/Cu_2_O Z‐scheme heterojunctions, the electron transfer process is depicted in the schematic diagram.

Following the successful fabrication of GQD/Cu_2_O, we delved into studying how the heterojunction enhances sonodynamic properties. The heterojunctions showed an enhanced ability to generate ROS, as seen in **Figure** **2**a,b. This was evident in the greater reduction of the absorption of DPBF compared to single‐component Cu_2_O. We also detected whether GQDs could generate ROS under US irradiation, which revealed no significant change of the absorption spectrum of GQDs in the presence of DPBF (Figure 2c). When comparing the ability to generate ROS, we calculated the rate constant of ROS production and observed that the heterojunction group showed a higher rate constant (Figure 2d). It is well known that the degradation of DPBF can be induced by the generation of ^1^O_2_ and O_2_ˉ^•^, which were both the types of ROS. Our investigation into the types of ROS generated led us to conduct ESR measurements specifically aimed at detecting ^1^O_2_. ESR spectra in Figure 2e revealed three resonance peaks of ^1^O_2_, confirming the generation of ^1^O_2_ during the sonodynamic reaction catalyzed by GQD/Cu_2_O. Furthermore, the GQD/Cu_2_O group showed stronger resonance peaks of ^1^O_2_ when contrasted with the other groups. Following that, we carried out fluorescence spectrum measurements on GQD/Cu_2_O, Cu_2_O, and GQDs using DHR123 as the probe, which has the capability to specifically detect O_2_ˉ^•^. As depicted in Figure 2f,g, the O_2_ˉ^•^ production can be detected in the GQD/Cu_2_O and Cu_2_O groups, which exhibited the enhanced fluorescence of DHR123. Similar to ^1^O_2_ generation efficiency, the higher O_2_ˉ^•^ production ability was observed in the heterojunction group. In contrast, insignificant O_2_ˉ^•^ generation was observed in the GQD group (Figure S5, Supporting Information), indicating that the single GQDs could not generate ROS under US irradiation. Through the energy transfer and electron transport processes, GQD/Cu_2_O heterojunctions were able to generate ^1^O_2_ and O_2_ˉ^•^. Furthermore, we also detected whether the GQD/Cu_2_O‐mediated sonodynamic mechanism involved the •OH generation, which was similar to the previous report.^[^ ^56^ ^]^ We utilized TMB as a •OH probe to determine the generation of •OH by GQD/Cu_2_O in the presence of US irradiation. As presented in Figure S6 (Supporting Information), no significant •OH production can be detected in GQD/Cu_2_O, Cu_2_O, or GQD in the presence of US irradiation, demonstrating that GQD/Cu_2_O‐mediated sonodynamic mechanisms did not involve the •OH generation.

We then delved into multiple band structure measurements to uncover the enhanced sonodynamic mechanisms of heterojunctions. The absorption spectrum data was used to initially measure the bandgap of single‐component GQDs and Cu_2_O. As shown in Figure 2h, both GQDs and Cu_2_O have a calculated bandgap of 2.2 eV, indicating that their narrow bandgap property. After constructing the energy band structure model, we then determined the valence band potential (*E*_VB_) of both GQDs and Cu_2_O. Figure 2i,j exhibited that the *E*_VB_ of GQDs and Cu_2_O was determined to be 0.58 and 1.35 eV, respectively, according to the VB‐XPS spectrum measurements. Hence, based on the values of *E*_VB_ and *E*_g_, the potential of the conduction band (*E*_CB_) for GQDs and Cu_2_O was determined to be −0.85 and −1.62 eV, respectively. Given the surface charge characteristics of GQDs and Cu_2_O, the built‐in electric field within heterojunctions was directed from GQDs to Cu_2_O (Figure 2k). It is likely that the improved sonodynamic efficacy of GQD/Cu_2_O was due to the establishment of Z‐scheme heterojunctions, which prevented the recombination of electron‐hole pairs.

**Figure 3:**
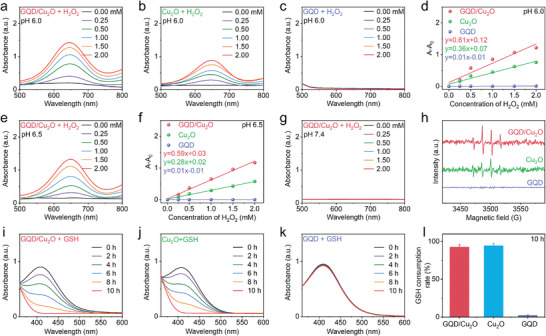
a–g) Measurements and comparison of •OH generation of GQD, Cu_2_O, and GQD/Cu_2_O at pH 6.0, 6.5, and 7.4. h) ESR spectra of GQD, Cu_2_O, and GQD/Cu_2_O at pH 6.0. i–l) Measurements of GSH consumption of GQD, Cu_2_O, and GQD/Cu_2_O. Data are presented as the mean ± SD. (*n* = 3).

We then investigated whether the Fenton‐like reaction activity of Cu_2_O would be augmented by the coating of GQDs. By observing the heightened absorbance of TMB at 652 nm, it was confirmed that •OH was produced in the presence of GQD/Cu_2_O or Cu_2_O at pH 6.0 (**Figure** **3**a,b). In the case of single‐component GQDs, there was no noticeable absorption change observed (Figure 3c), indicating that GQDs did not exhibit chemodynamic activity. Moreover, there was a noticeable difference in the rate constant of •OH production between the heterojunction group (0.61 min^−1^) and the single‐component Cu_2_O group (0.36 min^−1^) (Figure 3d). Apart from pH 6.0, our research also delved into the Fenton‐like reaction efficiency of heterojunctions at pH 6.5 and 7.4. Figure 3e demonstrated that GQD/Cu_2_O has the ability to generate •OH at pH 6.5, as indicated by the significant rise in absorbance at 652 nm. GQD/Cu_2_O also demonstrated higher •OH production efficiency at pH 6.5 in comparison to Cu_2_O alone (Figure S7, Supporting Information). Additionally, the behavior of pH‐dependent •OH generation by GQD/Cu_2_O and Cu_2_O showed a higher rate constant of •OH generation at pH 6.0 in comparison to pH 6.5 (Figure 3d,f). Moreover, it is crucial to point out that there is no evident formation of •OH in GQD/Cu_2_O at pH 7.4 (Figure 3g). Additionally, the absence of detectable •OH production in Cu_2_O at pH 7.4 (Figure S8, Supporting Information) may be attributed to the timing of the evaluation of Cu2O's chemodynamic activity at pH 7.4, which was carried out before its degradation. Figure 3h exhibited that the four characteristic peaks corresponded to •OH were detected in the heterojunctions, clearly confirming the generation of •OH.

In addition to Cu^+^‐mediated Fenton‐like reaction, we then explored whether GQD/Cu_2_O and Cu_2_O possessed the GSH depletion ability. As depicted in Figure 3i, the significantly decreased characteristic absorption peak of DTNB can be observed in GQD/Cu_2_O solution after incubating for 10 h, indicating that GQD/Cu_2_O can oxidize GSH to GSSG. In addition, the GSH consumption ability of Cu_2_O nanocubes was comparable to that of GQD/Cu_2_O heterojunctions (Figure 3j), which could be ascribed to the Cu^2+^‐mediated GSH depletion. For the single‐component GQDs, no obvious decrease of the characteristic absorption peak can be detected (Figure 3k). Furthermore, the data indicated that GQD/Cu_2_O consumed ≈93% of GSH, as shown in Figure 3l and Figure S9 (Supporting Information).

**Figure 4:**
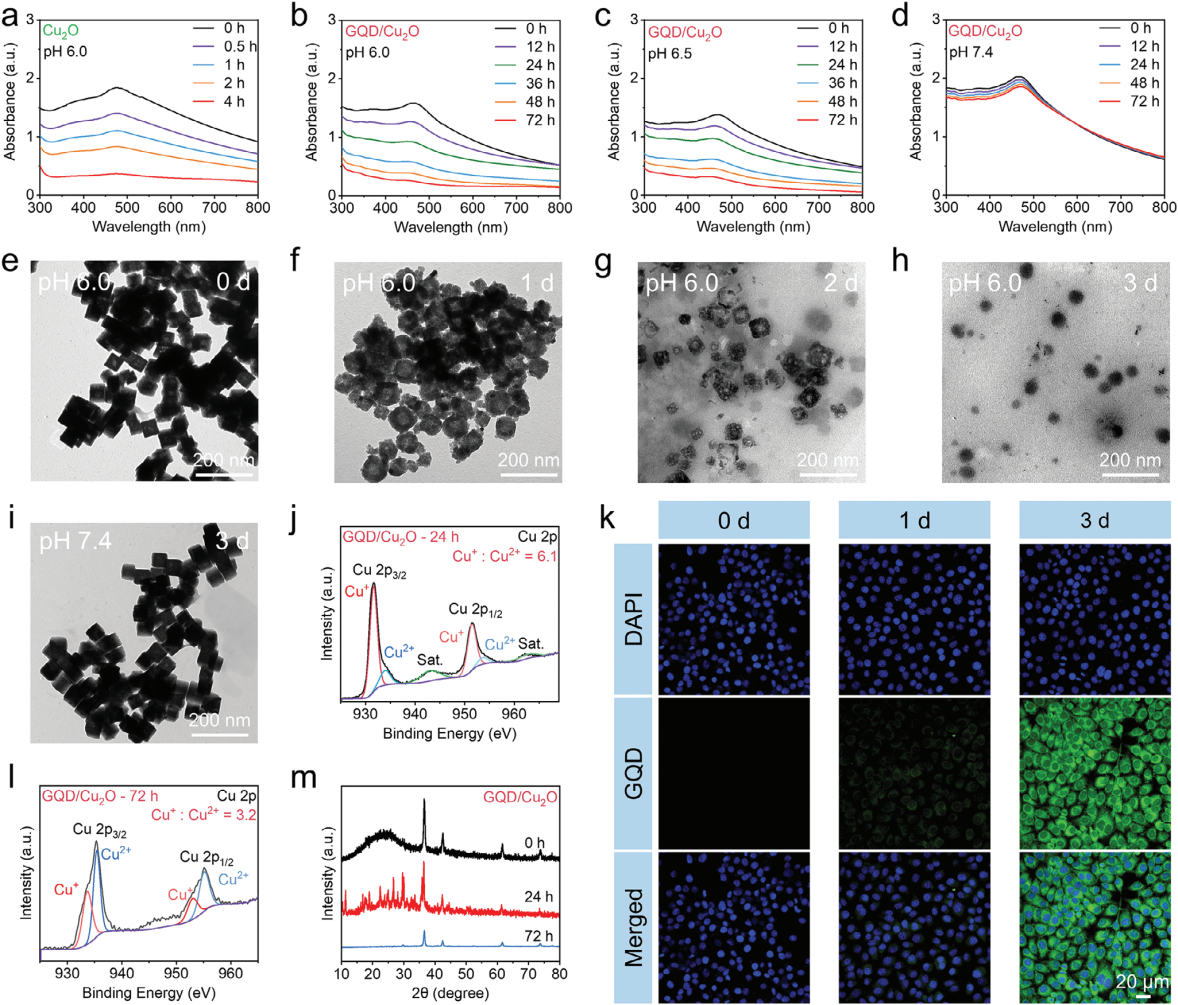
a–d) Absorption spectra of Cu_2_O and GQD/Cu_2_O after incubation at varied pH. e–m) TEM, high‐resolution Cu 2p spectra, XRD patterns of GQD/Cu_2_O after incubation at varied pH for different times. k) Confocal images of 4T1 cells treated with GQD/Cu_2_O for different times.

We then investigated the degradation behaviors of Cu_2_O nanocubes before and after GQD deposition to clarify the protective effect of GQDs on the degradation rate of Cu_2_O. As depicted in **Figure** **4**a, the sharply decreased absorption ranged from 300 to 800 nm was clearly observed in Cu_2_O nanocubes after incubation of only 4 h, suggesting that the fast degradation behaviors of Cu_2_O. Additionally, we took photographs of the Cu_2_O solution at pH 6.0 after different incubation times and observed a noticeable fading in color after 4 h (Figure S10, Supporting Information). The lightened color of the Cu_2_O solution after 4 h of incubation, similar to pH 6.0, indicated that Cu_2_O nanocubes can degrade in both acidic and normal physiological conditions. Figure S11 (Supporting Information) revealed that the absorption of Cu_2_O nanocubes exhibited significant decrease during the degradation process at pH 6.5 and 7.4, which was similar to that at pH 6.0. TEM images of Cu_2_O nanocubes after storing different times were also captured to assess the degradation behaviors of Cu_2_O nanocubes at varied pH. Figure S12 (Supporting Information) exhibited that the size of Cu_2_O nanocubes decreased after storing for 4 h at pH 6.0 and 7.4, forcefully demonstrating the complete degradation of Cu_2_O nanocubes. We further evaluated the chemodynamic property of Cu_2_O after degradation. As depicted in Figure S13 (Supporting Information), the obvious •OH generation was detected in Cu_2_O after degradation of 4 h, suggesting that the Fenton‐like reaction of Cu_2_O can also be induced at pH 7.4 after degradation. These results suggested that Cu_2_O can be degraded in normal physiological conditions before reaching the tumor sites, which makes it unable to achieve efficient SDT and CDT in tumor tissues.

After depositing GQDs, we then investigated the change of degradation behaviors of Cu_2_O nanocubes using optical measurements, TEM, XRD, XPS, and confocal microscope. The significantly reduced degradation behaviors of Cu_2_O nanocubes after loading GQDs can be observed in the absorption spectrum (Figure 4b,c), which revealed that the absorption of GQD/Cu_2_O heterojunctions after storing at pH 6.0 and 6.5 slowly decreased within 72 h. Moreover, insignificant change of the absorption spectrum of GQD/Cu_2_O heterojunctions can be detected after storing for 72 h at pH 7.4 (Figure 4d), illustrating that GQD/Cu_2_O cannot be degraded in normal physiological conditions. As presented in Figure S14 (Supporting Information), no obvious color change can be observed in GQD/Cu_2_O solution after storing for 72 h at pH 7.4, while the solution color became transparent at pH 6.0. The slow degradation behaviors of GQD/Cu_2_O were also verified by their TEM images, which exhibited a gradually collapsed structure of heterojunctions as the incubation time prolonging at pH 6.0 (Figure 4e–h). Notably, no significant change of the structure of GQD/Cu_2_O heterojunctions was detected after storing for 72 h at pH 7.4 (Figure 4i), clearly demonstrating the pH‐responsive degradation behaviors. The structural changes of heterojunctions during the degradation were then investigated by XPS and XRD characterizations to unveil the degradation mechanism. The results depicted in Figure 4j–l indicated a substantial decrease in the ratio of Cu^+^ to Cu^2+^ with increasing incubation time, pointing toward the oxidation of Cu^+^ to Cu^2+^ within GQD/Cu2O during the degradation process. For XRD patterns, the diffraction peaks of Cu_2_O in heterojunctions obviously decreased after incubation of 72 h. In addition to Cu_2_O, the broad diffraction peak of GQDs at ≈25° decreased significantly after 24 h of incubation, and completely disappeared after 72 h of incubation (Figure 4m), demonstrating that GQD could be completely released from GQD/Cu_2_O. Considering the fluorescence features and the obvious quenching effect of GQDs after loading on the surface of nanocubes (Figure S15, Supporting Information), we further evaluated the degradation performance of heterojunctions by detecting the fluorescence recovery of GQDs. Figure S16 (Supporting Information) exhibited that the fluorescence of GQD/Cu_2_O was enhanced after storing for 3 days at pH 6.0, while no significant fluorescence recovery of GQDs can be detected at pH 7.4. The GQD release was also detected by the confocal microscope, which exhibited the significant green fluorescence in 4T1 cells after incubation of 3 days (Figure 4k).

**Figure 5:**
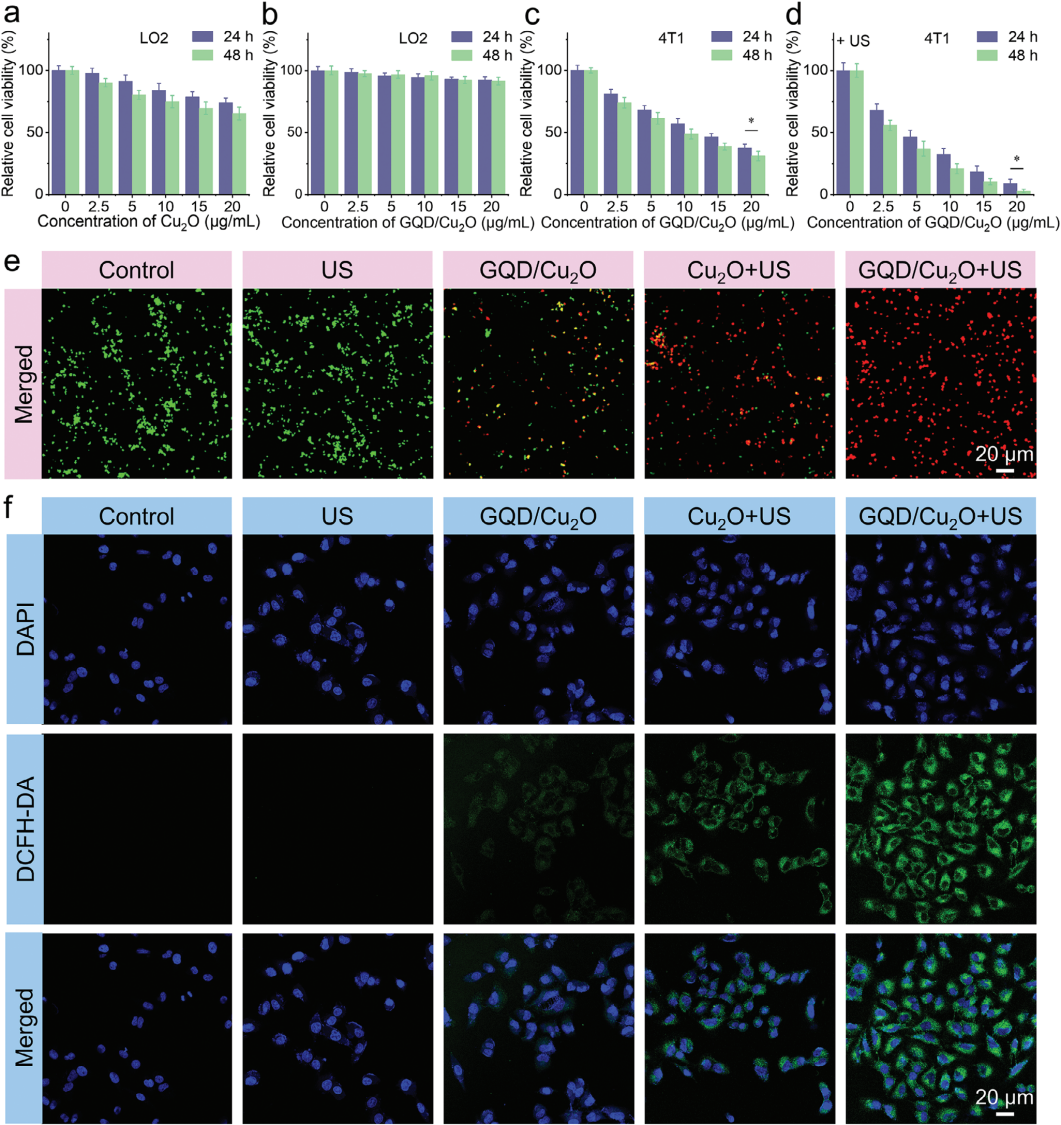
a–d) Measurements of the cytotoxicity of Cu_2_O and GQD/Cu_2_O through SDT, CDT, and cuproptosis. e,f) Measurements of antitumor efficacy and mechanism of GQD/Cu_2_O against 4T1 cells. Statistical significance between the two groups is calculated with a two‐tailed Student's *t*‐test. Data are presented as the mean ± SD. (*n* = 5). *p < 0.05.

Following the demonstration of the pH‐dependent degradation behaviors of GQD/Cu_2_O heterojunctions, we examined their effectiveness in combating tumors at a cellular level. The successful internalization of heterojunctions was initially confirmed by examining confocal images, which showed a prominent red fluorescence signal of ICG‐labeled GQD/Cu_2_O inside the cytoplasm of 4T1 cells (Figure S17, Supporting Information). The biocompatibility of GQD/Cu_2_O and Cu_2_O was evaluated by MTT assay. The decreased cell viability of the normal LO2 cells was detected after treating Cu_2_O nanocubes (**Figure** **5**a), indicating that Cu_2_O can degrade in the normal physiological conditions to release Cu^+^. 4T1 cells also showed a cytotoxic effect of Cu_2_O, indicating that Cu_2_O can degrade in acidic conditions (Figure S18, Supporting Information). No significant cytotoxicity of GQD/Cu_2_O against LO2 cells was detected (Figure 5b), demonstrating that the deposition of GQDs could avoid the degradation of Cu_2_O. However, GQD/Cu_2_O demonstrated clear cytotoxicity against 4T1 cells under identical conditions (Figure 5c), indicating that the released Cu^+^ may trigger chemodynamic activity and cuproptosis effect. Moreover, the severe cell death phenomenon of 4T1 cells treated with GQD/Cu_2_O in the presence of US irradiation was observed (Figure 5d). Moreover, the combination of GQD/Cu_2_O + US exhibited significantly greater cytotoxicity against 4T1 cells compared to the individual Cu_2_O nanocubes (Figure S19, Supporting Information). It is worth noting that single GQDs can lead to a significant decrease in cell viability of LO2 and 4T1 cells (Figure S20, Supporting Information), demonstrating the antitumor effectiveness of GQD‐mediated chemotherapy through inhibiting the activity of topoisomerase I/II and thereby causing DNA damage.^[^ ^55^ ^]^ However, the GQD‐induced cytotoxicity against LO2 cells was complete disappearance after depositing on Cu_2_O nanocubes (Figure 5b), which might be attributed to the difficulty of GQD release from heterojunctions to trigger DNA damage.

Apart from conducting the MTT assay, we also performed live/dead staining to evaluate the anti‐tumor efficacy of GQD/Cu_2_O in SDT, CDT, cuproptosis, and chemotherapy. The results in Figure 5e revealed that the GQD/Cu_2_O + US group exhibited the strongest red fluorescence signal, suggesting that the combination therapy effectively killed almost all 4T1 cells. It is worth noting that the GQD/Cu_2_O + US group showed the most intense green fluorescence signal when compared to the other groups (Figure 5f). This served as evidence that a majority of ROS was produced by GQD/Cu_2_O through the combined effects of SDT and CDT.

**Figure 6:**
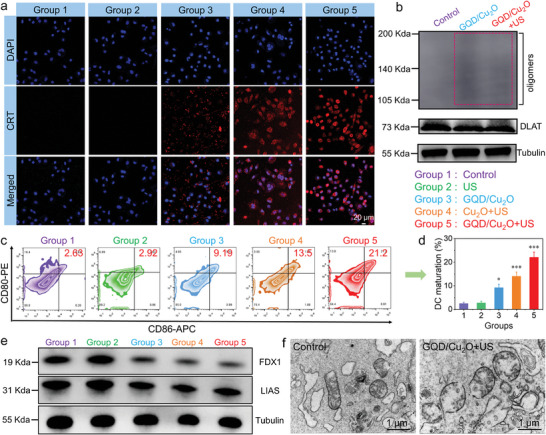
a) CRT levels in 4T1 cells were measured following various treatments. b) Following different treatments, Western blot analysis was used to examine the presence of DLAT and DLAT oligomers in 4T1 cells. c,d) By utilizing flow cytometry, we determined the expression of CD80 and CD86 in DCs. e) WB analysis of FDX1 and LIAS in 4T1 cells after different treatments. f) Bio‐TEM images of 4T1 cells before and after GQD/Cu_2_O + US treatment. Statistical significance between the experimental group and the control group is calculated with a two‐tailed Student's *t*‐test. Data are presented as the mean ± SD. (*n* = 3). *p < 0.05 and ***p < 0.001.

After showing the effectiveness of GQD/Cu_2_O in fighting tumors, we proceeded to conduct a set of experiments to uncover how they work against cancer. Taking into consideration the pH‐dependent release behaviors of Cu ions, we explored the influence of GQD/Cu_2_O on cuproptosis in cancer cells. Cuproptosis is recognized to be particularly effective against cancer cells exhibiting robust mitochondrial respiration, as exemplified by 4T1 cells (**Figure** **6**a).^[^ ^27^ ^]^ Consequently, we opted for 4T1 cells as the subject of our study to examine the impact of GQD/Cu_2_O on cuproptosis. When Cu ions interact with DLAT, it has the ability to trigger the DLAT oligomerization.^[^ ^57^ ^]^ Figure 6b exhibited that the obvious aggregation of DLAT was detected in 4T1 cells after treating with GQD/Cu_2_O, demonstrating that cuproptosis was successfully induced by GQD/Cu_2_O after degradation. Like GQD/Cu_2_O alone, the treatment of GQD/Cu_2_O + US also triggered the aggregation of DLAT. Figure 6e illustrated that the levels of LIAS and FDX1 were reduced in 4T1 cells when exposed to GQD/Cu_2_O alone, Cu_2_O + US, and GQD/Cu_2_O + US. Moreover, the GQD/Cu_2_O alone, Cu_2_O + US, and GQD/Cu_2_O + US groups showed a notable reduction in ATP levels in 4T1 cells, as depicted in Figure S21 (Supporting Information), indicating the ability of released Cu^+^ to impede ATP production through mitochondrial damage. The mitochondrial damage caused by GQD/Cu_2_O‐mediated cuproptosis was further investigated by the bio‐TEM characterization. Figure 6f exhibited that the obvious mitochondrial damage can be detected in 4T1 cells after incubation of GQD/Cu_2_O + US. The membrane potential change of mitochondria in 4T1 cells was then investigated using JC‐1 as the probe to further confirm the mitochondrial damage. Upon exposure to GQD/Cu_2_O, a pronounced green fluorescence was observed in 4T1 cells (Figure S22, Supporting Information), elucidating the dysfunctional mitochondria induced by GQD/Cu_2_O‐mediated cuproptosis. Furthermore, the GQD/Cu_2_O + US group demonstrated the most intense green fluorescence signal, indicating that GQD/Cu_2_O‐mediated cuproptosis could enhance the therapeutic outcomes of SDT and CDT by inducing significant mitochondrial dysfunction. It was found that GQD/Cu_2_O heterojunctions played a key role in promoting cuproptosis, which in turn boosted the effectiveness of cell death through SDT/CDT‐induced apoptosis.

Following the successful demonstration of GQD/Cu_2_O‐induced apoptosis and cuproptosis, we then explored the cell death mechanism. Reports have indicated that the production of ROS and the triggering of cuproptosis are capable of starting ICD effects.^[^ ^50^, ^58^, ^59^, ^60^, ^61^, ^62^, ^63^, ^64^ ^]^ We initially used semi‐quantitative analysis to examine the expression of CRT in 4T1 cells. The strongest CRT signal was observed in the GQD/Cu_2_O + US group (Figure 6a), implying that the optimal therapeutic effects of GQD/Cu_2_O under US irradiation. In addition to confocal microscope, we further utilized flow cytometry to detect the CRT exposure in 4T1 cells. The results from Figure S23 (Supporting Information) illustrated that the GQD/Cu_2_O + US group had the most CRT positive cells among all the groups, which was in line with the confocal imaging findings. Figure S24 (Supporting Information) revealed that the treatment of GQD/Cu_2_O + US possessed the highest HMGB1 level in 4T1 cells. The extracellular ATP level was higher in the GQD/Cu_2_O + US group compared to the other groups (Figure S25, Supporting Information). It is evident from the above findings that the potent ICD effects were brought about by the synergistic effects of ROS and cuproptosis.

As DAMPs may be released during GQD/Cu_2_O‐induced ICD effects and have the potential to bind to DCs, we used flow cytometry analysis to determine the proportion of mature DCs following various treatments. Upon introducing the supernatant from 4T1 cells subjected to different treatments to DCs, the levels of CD86 and CD80 expression on DCs were examined to establish the maturation level of DCs. In Figure 6c, it is evident that there is a higher proportion of CD80^+^CD86^+^ DCs in the GQD/Cu_2_O alone group as opposed to the control group. This indicated that the induction of ICD by GQD/Cu_2_O‐mediated CDT and cuproptosis can promote the maturation of DCs. Additionally, the GQD/Cu_2_O + US group showed the highest percentage of CD80^+^CD86^+^ DCs among all groups (Figure 6d).

**Figure 7:**
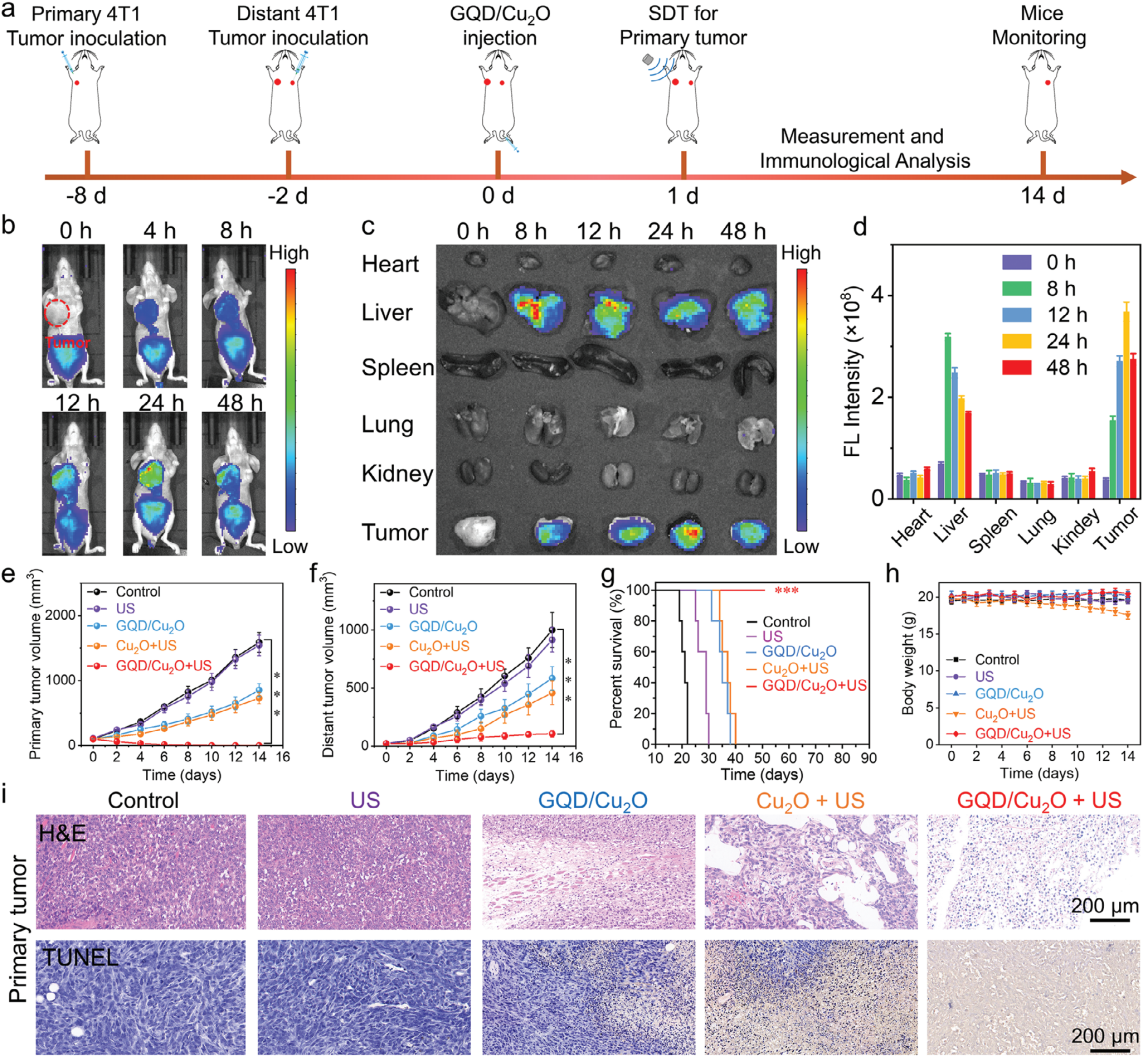
a) The schematic illustration showed the in vitro anticancer therapy procedures of GQD/Cu_2_O. b–d) The accumulation of GQD/Cu_2_O in tumor tissues. e–h) After receiving different treatments, the primary and distant tumor volume, survival rate, and body weight of mice were assessed. i) H&E and TUNEL staining of tumor tissues in different groups. Statistical significance between the experimental group and the control group is calculated with a two‐tailed Student's *t*‐test. Data are presented as the mean ± SD. (*n* = 5). ***p < 0.001.

To investigate the antitumor efficacy of GQD/Cu_2_O, a bilateral tumor model was established in mice and the administration of GQD/Cu_2_O in the presence of US irradiation was performed according to the tailored treatment plans (**Figure** **7**a). In Figure 7b and Figure S26 (Supporting Information), it can be observed that the fluorescence signal of ICG‐labeled GQD/Cu_2_O increased progressively in the tumor tissues, reaching its highest point 24 h after injection. The ex vivo NIR imaging in Figure 7c,d exhibited comparable results, confirming the efficient accumulation of GQD/Cu_2_O in tumor tissues. This phenomenon is likely due to the enhanced permeation and retention (EPR) effect. We chose this time window (1 day) because GQD/Cu_2_O accumulated the most in tumor tissue at 24 h post‐injection.

The antitumor efficacy of GQD/Cu_2_O was then evaluated by measuring the volume of primary and distant tumors. The therapeutic effect of GQD/Cu_2_O on the primary tumor, as depicted in Figure 7e, may be attributed to the Cu^+^‐induced CDT and cuproptosis effects. Furthermore, aside from targeting primary tumors, GQD/Cu_2_O also demonstrated effectiveness in treating distant tumors (Figure 7f). This suggested that GQD/Cu_2_O could potentially trigger an immune response by inducing ICD effects through ROS and cuproptosis mechanisms. Furthermore, the primary tumors in the GQD/Cu_2_O + US group were eradicated entirely within a span of 6 days. The GQD/Cu_2_O + US group exhibited nearly complete inhibition of distant tumor growth, outperforming the GQD/Cu_2_O alone group. As illustrated in Figure 7i, S27, S28, the primary and distant tumor cells in GQD/Cu_2_O + US group were almost all apoptosis or necrosis. Additionally, the mice treated with GQD/Cu_2_O + US were able to survive for more than 50 days (Figure 7g), indicating that the cuproptosis‐enhanced SDT/CDT approach could induce strong immunotherapy for the extension of mice lifespan. Figure S29 (Supporting Information) exhibited that the strongest ROS signal was detected in GQD/Cu_2_O + US group, suggesting that the cuproptosis‐amplified SDT/CDT generated the most ROS compared with the other groups. The results mentioned above clearly show that the treatment with GQD/Cu_2_O + US successfully eliminated the primary tumors and triggered abscopal effects that halted the growth of distant tumors.

After successfully showing the therapeutic effectiveness of GQD/Cu_2_O, we proceeded to study the immune reactions triggered by GQD/Cu_2_O to understand the in vivo anti‐tumor mechanism. The GQD/Cu_2_O + US group exhibited the strongest green fluorescence signal in Figure S30 (Supporting Information), suggesting the highest level of CRT exposure. Furthermore, it was observed that the released HMGB1 level in the GQD/Cu_2_O + US group was significantly higher compared to the other groups (Figure S31, Supporting Information). This suggested that GQD/Cu_2_O‐based tumor therapy has the potential to induce a more robust ICD effect through the generation of ROS and initiation of cuproptosis. The results depicted in Figure S32 (Supporting Information) showed that treatment with GQD/Cu_2_O notably reduced ATP levels in tumor tissues, indicating the strong immune responses triggered by GQD/Cu_2_O when exposed to ultrasound irradiation.

**Figure 8:**
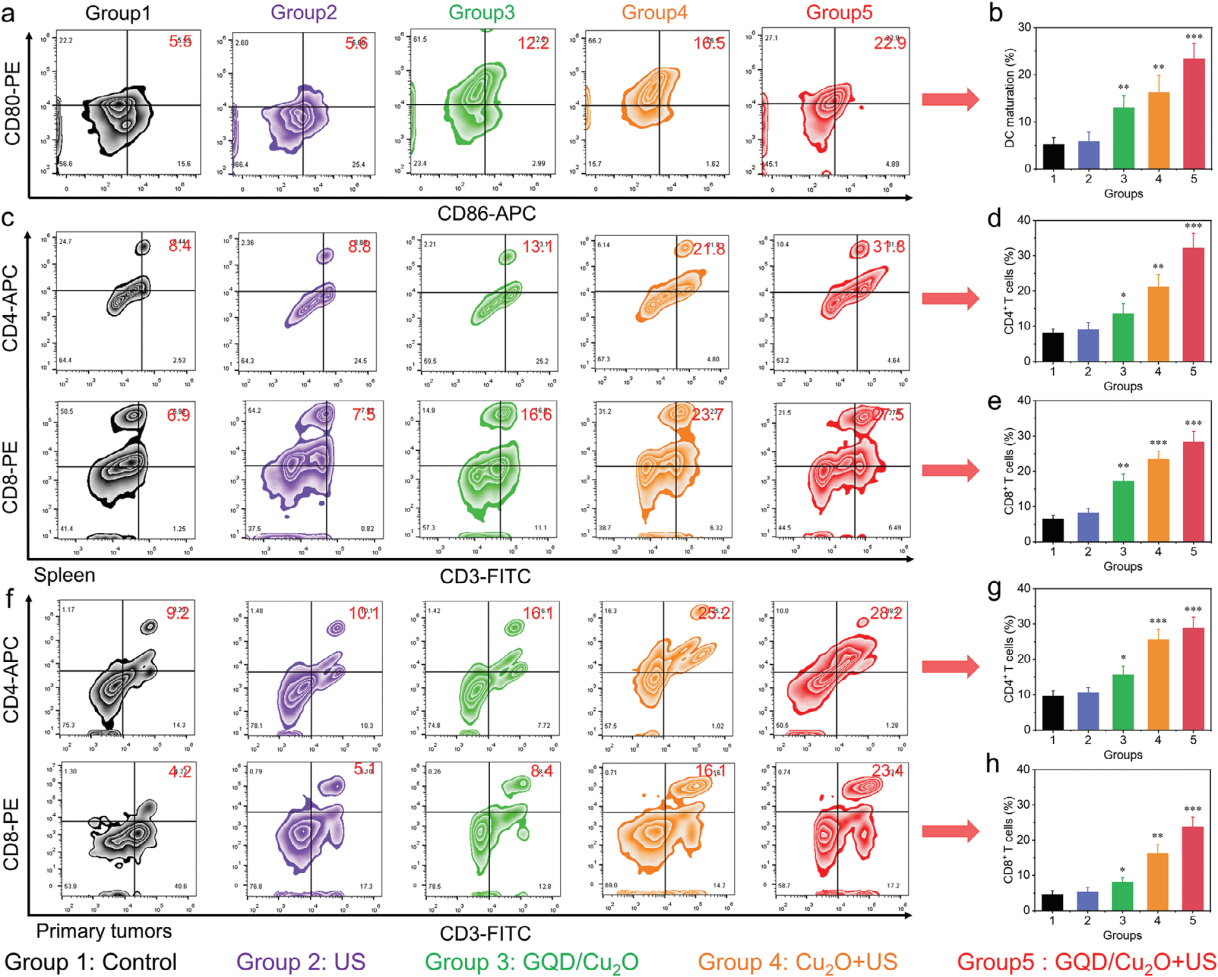
a,b) Assessment of CD80 and CD86 expression in lymph nodes following various treatments. c–h) The expression of CD4^+^CD8^+^ T cells in both the spleen and primary tumors will be evaluated after receiving different treatments. Statistical significance between the experimental group and the control group is calculated with a two‐tailed Student's *t*‐test. Data are presented as the mean ± SD. (*n* = 3). *p < 0.05, **p < 0.01, and ***p < 0.001.

Once the remarkable ICD effects induced by GQD/Cu_2_O combination therapy were confirmed, the next step was to investigate whether the maturation of DCs could be triggered by combining GQD/Cu_2_O treatment with US irradiation. The results from **Figure** **8**a suggested that the maturation of DCs was not markedly impacted by either PBS or US irradiation treatments, potentially because there was little ROS generation. Conversely, the group treated exclusively with GQD/Cu_2_O exhibited a growth in the number of mature DCs that displayed CD80 and CD86 (Figure 8b), indicating the production of ROS and the triggering of cuproptosis. A noteworthy increase was observed in the populations of CD80^+^CD86^+^ mature DCs in the GQD/Cu_2_O + US group, as opposed to the other groups. Following the verification of the enhanced impacts of ICD and the hastened maturation of DCs with the use of SDT/CDT and cuproptosis employing GQD/Cu_2_O, we then investigated the possible stimulation of T cells by GQD/Cu_2_O‐mediated combination therapy. Upon exposure to GQD/Cu_2_O + US treatment, a higher percentage of CD4^+^CD8^+^ T cells in the spleen was observed in comparison to the other groups (Figure 8c–e). Following treatment with GQD/Cu_2_O and US irradiation, there was a higher number of CD4^+^CD8^+^ T cells present in both primary and distant tumors (Figure 8f–h; Figure S33, Supporting Information). This suggested that the use of SDT/CDT in conjunction with cuproptosis induced by GQD/Cu_2_O can trigger robust immune responses and aid in the activation of immunotherapy. Furthermore, we utilized immunofluorescence staining to detect the infiltration of CD4^+^ and CD8^+^ T cells in the primary and distant tumors. The GQD/Cu_2_O + US group exhibited the strongest fluorescence signals of CD4^+^CD8^+^ T cells in the primary and distant tumors, in contrast to the other groups (Figures S34 and S35, Supporting Information). These findings conclusively demonstrated that the release of Cu ions and GQDs in tumors could trigger a cascade amplification of ROS generation, ultimately leading to efficient cuproptosis and GQD‐mediated DNA damage.

**Figure 9:**
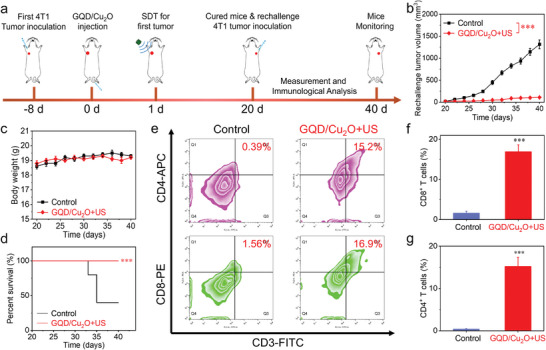
a) Diagram depicting the GQD/Cu_2_O‐based combination therapy for generating anticancer immune memory and preventing cancer recurrence. b–d) Analyze the tumor volume, body weight, and survival rate of mice post different treatments by rechallenging them. e–g) The expression of CD4^+^CD8^+^ T cells in the rechallenged tumors will be evaluated post various treatments. Statistical significance between the experimental group and the control group is calculated with a two‐tailed Student's *t*‐test. Data are presented as the mean ± SD. (*n* = 5). ***p < 0.001.

A crucial aspect of adaptive immunity is the immunological memory response, which enables the immune system to recognize and defend against previously encountered pathogens, ensuring enduring immunity. We then constructed a rechallenging tumor model to investigate the long‐term immune‐memory therapeutic effects of GQD/Cu_2_O‐mediated combination therapy. As depicted in **Figure** **9**a, after the first round of GQD/Cu_2_O‐mediated combination therapy was completed, the mice were rechallenged with 4T1 cells on the opposite side of the axilla in mice on day 20. Meanwhile, five mice, matched in terms of sex and age, were used as controls and were inoculated with the same number of 4T1 tumor cells. Figure 9b exhibited that the tumor volume in the control group increased rapidly after inoculation. For comparison, the significantly inhibited tumor progression was detected in the GQD/Cu_2_O + US group, proving that exceptional immune memory responses were established after the GQD/Cu_2_O‐mediated combination therapy, which could provide long‐lasting protection for these mice from tumor relapse. No fluctuation of body weight can be detected in the two groups (Figure 9c), demonstrating the safety of GQD/Cu_2_O‐mediated combination therapy. The survival time of mice in GQD/Cu_2_O + US group was more than 40 days (Figure 9d), indicating that the excellent immune memory responses induced by GQD/Cu_2_O‐mediated combination therapy could prolong the lifetime of mice. We then investigated the potential activation of T cells by GQD/Cu_2_O in conjunction with US irradiation in rechallenging tumor models. The higher proportion of CD4^+^CD8^+^ T cells in the rechallenging tumor was detected in the GQD/Cu_2_O + US group compared with the control group (Figure 9e–g), suggesting the potent immune responses.

Ensuring the biosafety of nanomedicine is paramount in guaranteeing their clinical efficacy for tumor treatment. Therefore, we examined the biosafety of GQD/Cu_2_O and its potential for tumor treatment. The single‐component Cu_2_O group experienced significant weight loss during the treatment periods, which was attributed to the release of Cu^+^ triggering cuproptosis in normal tissues (Figure 7h). The unregulated discharge of Cu^+^ in regular tissues could lead to serious safety concerns and impede its practicality in clinical settings. Once GQDs were loaded, there were no significant changes in the body weight of the mice, showing that the deposition of GQDs effectively prevented the degradation of Cu_2_O in normal tissues. The absence of any noticeable pathological abnormalities in the major organs of the GQD/Cu_2_O + US group, as demonstrated in Figure S36 (Supporting Information), indicated that GQD/Cu_2_O did not degrade in these organs to cause the Fenton‐like reaction and cuproptosis. Figure S37 (Supporting Information) illustrated that all markers of blood biochemistry and blood routine examination in the GQD/Cu_2_O + US group fell within normal concentration ranges. This indicated that GQD and Cu^+^ are not released from GQD/Cu_2_O to trigger SDT, CDT, cuproptosis, and DNA damage in normal tissues.

## 3 Conclusion

In summary, we utilize GQDs as a protection layer to regulate the degradation behaviors of Cu_2_O nanocubes. After deposition of GQDs, the non‐tumor‐specific degradation of Cu_2_O nanocubes is inhibited, which avoids the inevitable damage on normal tissues. On the basis of tumor‐specific degradation of Cu_2_O nanocubes, the merits of GQD/Cu_2_O heterojunctions for potential clinical translation can be summarized as follows. First, although the GQD/Cu_2_O heterojunctions will inevitably accumulate in normal tissues, Cu^+^ is unable to exert chemodynamic and cuproptosis functions because the degradation of heterojunctions only occurs in tumor tissues. Second, the chemodynamic activity and cuproptosis effect can be specifically activated in tumor tissues owing to the tumor‐specific release of Cu^+^. In addition, the cascade amplification of ROS production is realized by the Cu^2+^‐mediated GSH consumption. Third, the released GQDs could inhibit topoisomerase I/II and cause DNA damage to induce apoptosis for enhanced chemotherapy. Finally, the immunosuppressive TME can be reversed by the significantly amplified ROS level and high‐efficiency cuproptosis, which could trigger ICD effects to induce robust systemic immune responses.

## 4 Experimental Section

### Synthesis of Cu_2_O Nanocubes

After dissolving 0.05 g of SDS in 10 mL of DI water and stirring for 5 min, 100 µL of CuSO_4_ was added to the solution. Subsequently, 40 µL of NaOH was mixed into the solution. Consequently, the solution changed to a light bluish color, showing the presence of Cu(OH)_2_ precipitate. Ultimately, the mixture solution was supplemented with 0.25 mL of 0.2 M sodium ascorbate solution. Following a 5 min stirring period and a subsequent 10 min period of aging without disturbance to promote crystal growth, the color of the mixture solution transitioned to yellow, indicating the emergence of Cu2O nanocubes.

### Synthesis of GQDs

A facile microwave method was utilized to synthesize GQDs. In brief, julolidine (0.025) and oxalic acid (0.25 mL) were selected as the precursors and the reaction temperature was 200 °C. Once the reaction had taken place for 15 min, the GQDs were purified by repeatedly using ultrasonic extraction with petroleum ether.

### Synthesis of GQD/Cu_2_O Heterojunctions

When making GQD/Cu_2_O heterojunctions, 5 mL of GQDs (1 mg mL^−1^) were mixed with 1 mL of Cu_2_O nanocubes (1 mg mL^−1^). Then, the centrifugation (10 000 rpm, 10 min) was performed to remove the undeposited GQDs to obtain GQD/Cu_2_O heterojunctions.

### Characterization

TEM images of GQD, Cu_2_O, and GQD/Cu_2_O were acquired by a JEM‐2100F microscope. XRD measurements of GQD, Cu_2_O, and GQD/Cu_2_O were conducted by Rigaku 18 KW D/max‐2550. The valence of elements in GQD, Cu_2_O, and GQD/Cu_2_O were measured by XPS. Fluorescence spectrum was measured by Agilent Cary 5000 spectrophotometer. The Malvern Nano ZS90 analyzer was utilized to acquire the hydrodynamic diameter of GQD, Cu_2_O, and GQD/Cu_2_O.

### Sonodynamic Performance Measurements

For the detection of ROS generation, 60 µL DPBF was added to 3 mL of GQD, Cu_2_O, or GQD/Cu_2_O solution. The sample was exposed to US irradiation with a frequency of 50 kHz and power density of 1.0 W cm^2^ for a duration of 10 min. Subsequently, the absorption spectrum was examined to identify any variations in the DPBF levels at 418 nm.

For the detection of ^1^O_2_, 20 µL of 2,2,6,6‐tetramethylpiperide (TEMP) was added to 1 mL of GQD, Cu_2_O, or GQD/Cu_2_O solution (20 µg mL^−1^) and US irradiation was performed for 2 min. Electron spin resonance (ESR) spectrum was then carried out to determine the generation of ^1^O_2_.

For the detection of O_2_^−•^, dihydrorhodamine 123 (DHR123) was used as an O_2_^−•^ indicator. GQD, Cu_2_O, or GQD/Cu_2_O solution (20 µg mL^−1^) was mixed with DHR123 (10 mM) in DI water. The mixture solution was exposed to US irradiation for different times, and the emission spectra were detected immediately after each irradiation (E_x_: 500 nm).

### Chemodynamic Performance Measurements

The absorption spectrum was utilized to evaluate the chemodynamic property of GQD, Cu_2_O, or GQD/Cu_2_O by measuring the absorbance change of TMB at 652 nm during the catalytic reaction. We added 20 µL of TMB to the GQD, Cu_2_O, or GQD/Cu_2_O solution in the presence of H_2_O_2_ with various concentrations (0, 0.25, 0.50, 1.00, 1.50, and 2.00 mM), respectively.

### GSH Depletion Performance Measurements

The combination of GQD, Cu_2_O, or GQD/Cu_2_O with 1 mM GSH in a PBS solution (pH 7.4) was the first step. Following different stirring times, 300 µL of the mixture was combined with 2700 µL of PBS, and then 2 µL of DTNB (0.2 mM) was introduced. By examining the variations in the absorption peak of DTNB at 412 nm, it was able to calculate the GSH depletion potential of GQD, Cu_2_O, or GQD/Cu_2_O.

### TME‐Responsive Degradation of GQD/Cu_2_O Heterojunctions

For the assessment of degradation properties, Cu_2_O or GQD/Cu_2_O (20 µg mL^−1^) was dispersed in the buffer solution at pH 6.0, 6.5, or 7.4. Upon completion of stirring at different time intervals, the absorption spectra, TEM, XRD, and XPS of the Cu_2_O or GQD/Cu_2_O solution were examined.

### MTT Assay

LO2 and 4T1 cells from Haixing Biosciences (Suzhou, Jiangsu, China) was purchased. The two cells were seeded into the 96‐well plates (5×10^3^ cells). After cells were incubated with samples for 4 h, US irradiation was applied for in vitro SDT. After being exposed to US irradiation for 10 min, the cells were left to incubate for either 24 or 48 h. The cytotoxicity levels were then determined using a standard MTT assay, following the methodology described in an earlier publication.^[^ ^65^ ^]^

### In Vitro ROS Detection and Live/Dead Cell Staining

The ROS level in different groups was determined using DCFH‐DA as the ROS probe. All the images were acquired by confocal microscope (Olympus, Japan). When conducting live/dead cell staining, a fluorescence microscope (Olympus, Japan) to capture fluorescence images was employed.

### Western Blotting Assay

Cultured in a 6‐well plate at a density of 10^5^ cells well^−1^, 4T1 tumor cells were incubated with GQD, Cu_2_O, or GQD/Cu_2_O. Following a 24 h incubation period, the cell lysates were collected. After incubation with antibodies to DLAT (1:20 000, proteintech), LIAS (1:1000, Abcam), FDX1 (1:1000, Abcam), γ‐H2AX (Abcam), or α‐tubulin (1:1000, Abcam), the cell lysates were analyzed and detected with a chemiluminescent imaging system.

### In Vitro Detection of ICD Biomarkers

For the detection of HMGB1, CRT, and ATP, the 4T1 cells were treated with different groups, including control, US, GQD/Cu_2_O, Cu_2_O + US, and GQD/Cu_2_O + US. After collection, the supernatants were analyzed using the HMGB1 Elisa Kit in accordance with the provided instructions. Once subjected to various treatments, the intracellular ATP level in 4T1 cells was analyzed according to the provided protocol. In preparation for confocal imaging or flow cytometry, cells were first fixed with immunostaining fixative for 5 min and then blocked with immunostaining blocking buffer for 30 min. After being rinsed three times with Immunol staining wash buffer, the cells were next subjected to a 30 min incubation with the secondary antibody (Alexa Fluor 647) as recommended.

### In Vitro Evaluation of Mature DCs

BMDCs were derived from the humerus and tibia of Balb/c mice (6‐8 weeks old), and then incubated in the RPMI‐1640 medium (containing GM‐CSF and IL‐4) for 7 days. On the seventh day, BMDCs were incubated with a suspension of 4T1 cells that had been treated with different groups (control, US, GQD/Cu_2_O, Cu_2_O + US, and GQD/Cu_2_O + US). Following a 24 h incubation period, the suspended cells were collected. Flow cytometry was then employed to analyze the BMDCs.

### In Vivo NIR Fluorescence Imaging

It was purchased 5‐week‐old female Balb/c mice from SLAC Laboratory Animal in Shanghai, China. Under the authorization of the Institutional Animal Care and Use Committee of Shanghai University (SYXK 2019‐0020), all animal experiments were carried out.

For the subcutaneous tumor, ICG‐labeled GQD/Cu_2_O (0.2 mg kg^−1^) were intravenously injected into mice. Once intravenously injected at different times, NIR fluorescence images were obtained using an IVIS Lumina III in vivo Imaging System (PerkinElmer, USA).

### In Vivo Antitumor Effect in Bilateral Tumor Model

To establish a bilateral tumor model, mice were injected with 1 × 10^6^ 4T1 cells into the left and right axilla at day −8 and day −2, respectively. At day 0, when the tumor volume reached ≈100 and 20 mm^3^, respectively, mice were treated with PBS, Cu_2_O, or GQD/Cu_2_O through intravenous injection. The dose of Cu_2_O or GQD/Cu_2_O was 0.2 mg kg^−1^. The primary tumors were subjected to US irradiation (50 kHz, 1.0 W cm^2^) for 10 min on day 1. Subsequently, on day 2, the tumors were retrieved for H&E and TUNEL staining. By day 7, the primary and distant tumors, spleens, and lymph nodes were collected to examine ICD markers, DC maturation, and T cell activation.

### In Vivo Antitumor Effect in Rechallenging Tumor Model

To set up a rechallenging tumor model, mice were injected with 1 × 10^6^ 4T1 cells subcutaneously into the right axilla on day −8 to establish the primary tumors. After the first tumors reached a volume of ≈100 mm^3^, the mice were treated with GQD/Cu_2_O by intravenous injection. The dose of GQD/Cu_2_O was 0.2 mg kg^−1^. The first tumors were exposed to US irradiation for 10 min on day 1. Following this, on day 20, 1 × 10^6^ 4T1 cells were subcutaneously injected into the left axilla of five mice that had been cured, to create the rechallenging tumors. Controls consisted of five mice that were matched based on sex and age.

### Statistical Analysis

Mean ± standard deviation was used to present the data, and each experiment was carried out with a minimum of three independent replicates. Origin 2018 and Excel were utilized for all statistical analyses. A two‐tailed Student's *t*‐test was used to calculate the statistical significance between the two groups. * denotes a statistical significance (*p < 0.05, **p < 0.01, and ***p < 0.001) between the experimental data of two groups.

## Conflict of Interest

The authors declare no conflict of interest.

## Supporting information



Supporting Information

## Data Availability

The data that support the findings of this study are available from the corresponding author upon reasonable request.
