# Supplementary material for: Graphene Quantum Dot Sensitized Heterojunctions Induce Tumor‐Specific Cuproptosis to Boost Sonodynamic and Chemodynamic Enhanced Cancer Immunotherapy
Source: Adv Sci (Weinh). 2024 Dec 24;12(7):2410606. doi: 10.1002/advs.202410606 (PMC11831527; doi:10.1002/advs.202410606)
Supplement: Supplementary file 1 — Supporting Information [file ADVS-12-2410606-s001.docx]

Supporting Information

**Graphene Quantum Dot Sensitized Heterojunctions Induce Tumor-Specific Cuproptosis to Boost Sonodynamic and Chemodynamic Enhanced Cancer Immunotherapy**

*Lang Yan, Liang Chang, Yijun Tian, Jinyan Hu, Zhi Cao,* Xiang Guo,* Bijiang Geng**

L. Yan, Y. Tian

Department of Health Toxicology, Faculty of Naval Medicine, Naval Medical University, Shanghai, 200433, China

L. Chang

Department of Emergency and Critical Care, Shanghai Changzheng Hospital, Second Affiliated Hospital, Naval Medical University, Shanghai, 200003, China

J. Hu, B. Geng

School of Environmental and Chemical Engineering, Shanghai University, Shanghai 200444, China

E-mail: bjgeng1992@shu.edu.cn (B. Geng)

Z. Cao

Department of Urology, Changhai Hospital, Naval Medical University, Shanghai, 200433, China

E-mail: caozhi@smmu.edu.cn (Z. Cao)

X. Guo

Department of Orthopaedics, Shanghai Changzheng Hospital, Second Affiliated Hospital, Naval Medical University, Shanghai, 200003, China

E-mail: [gx9601074@smmu.edu.cn](mailto:gx9601074@smmu.edu.cn) (X. Guo)


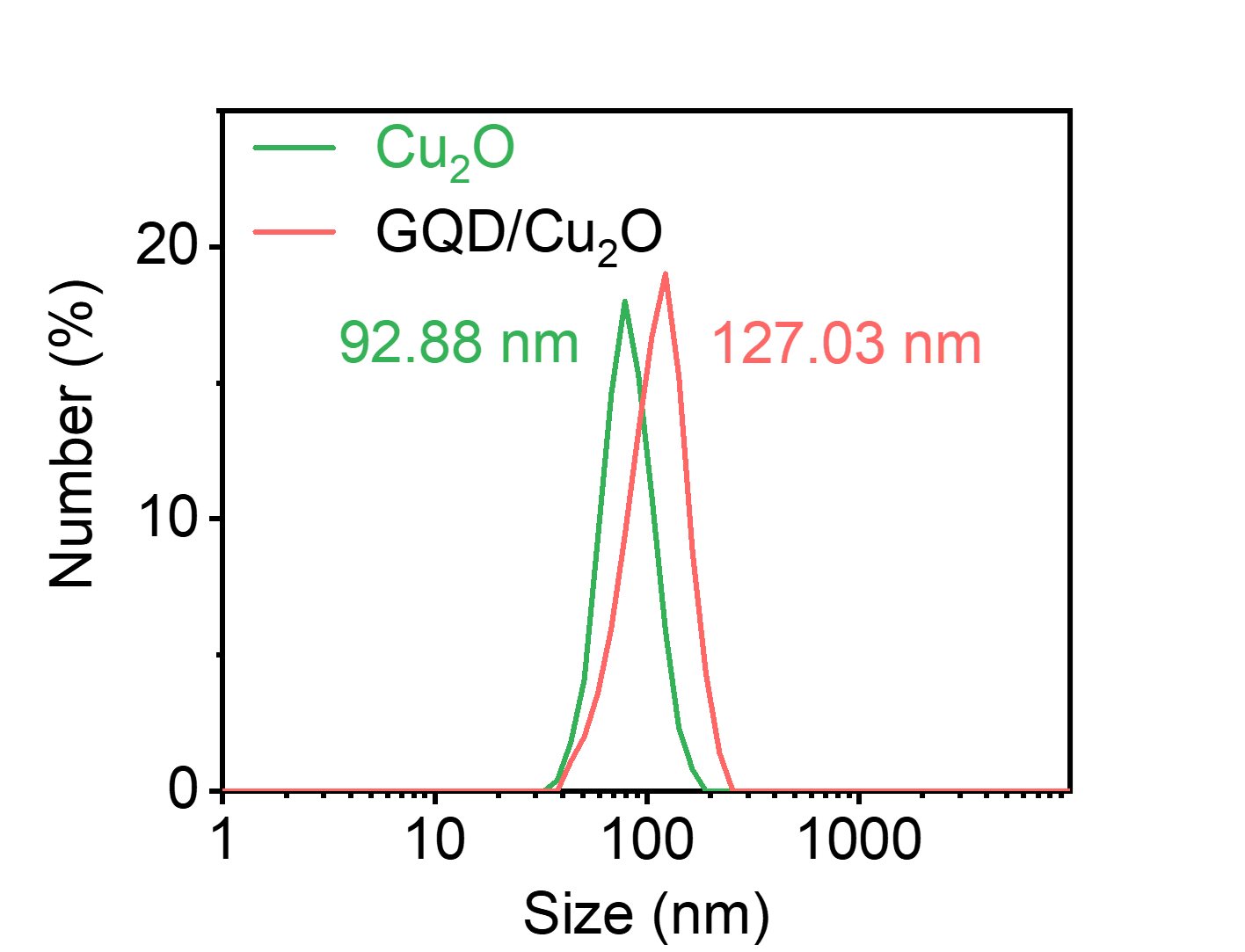


**Figure S1.** Hydrodynamic diameter of Cu_2_O and GQD/Cu_2_O.


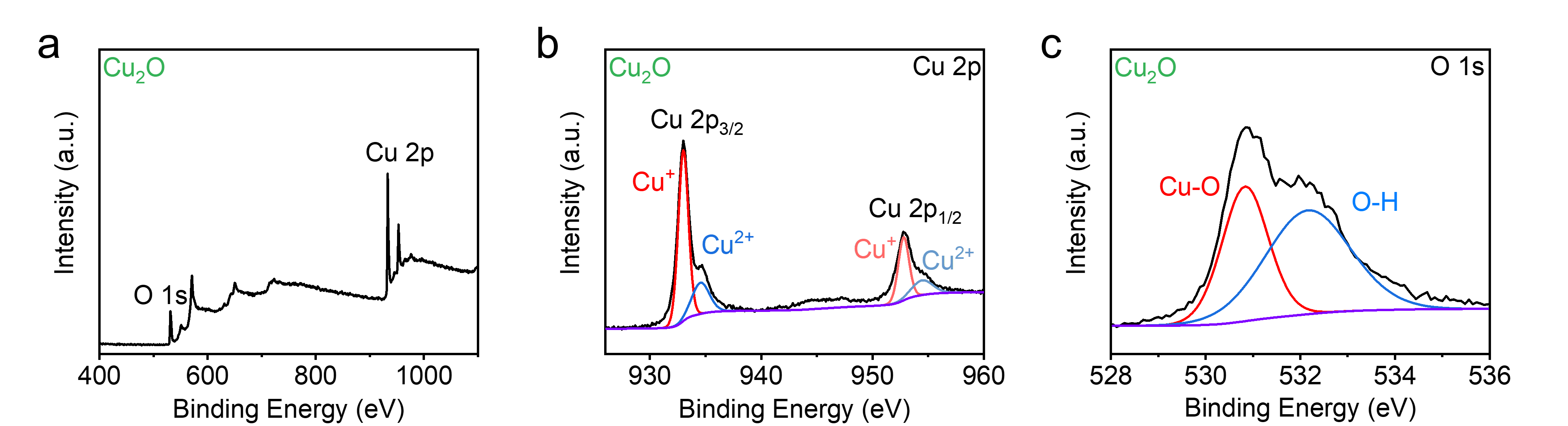


**Figure S2.** Survey XPS spectra (a), high-resolution Cu 2p (b) and O 1s (c) spectra of Cu_2_O.


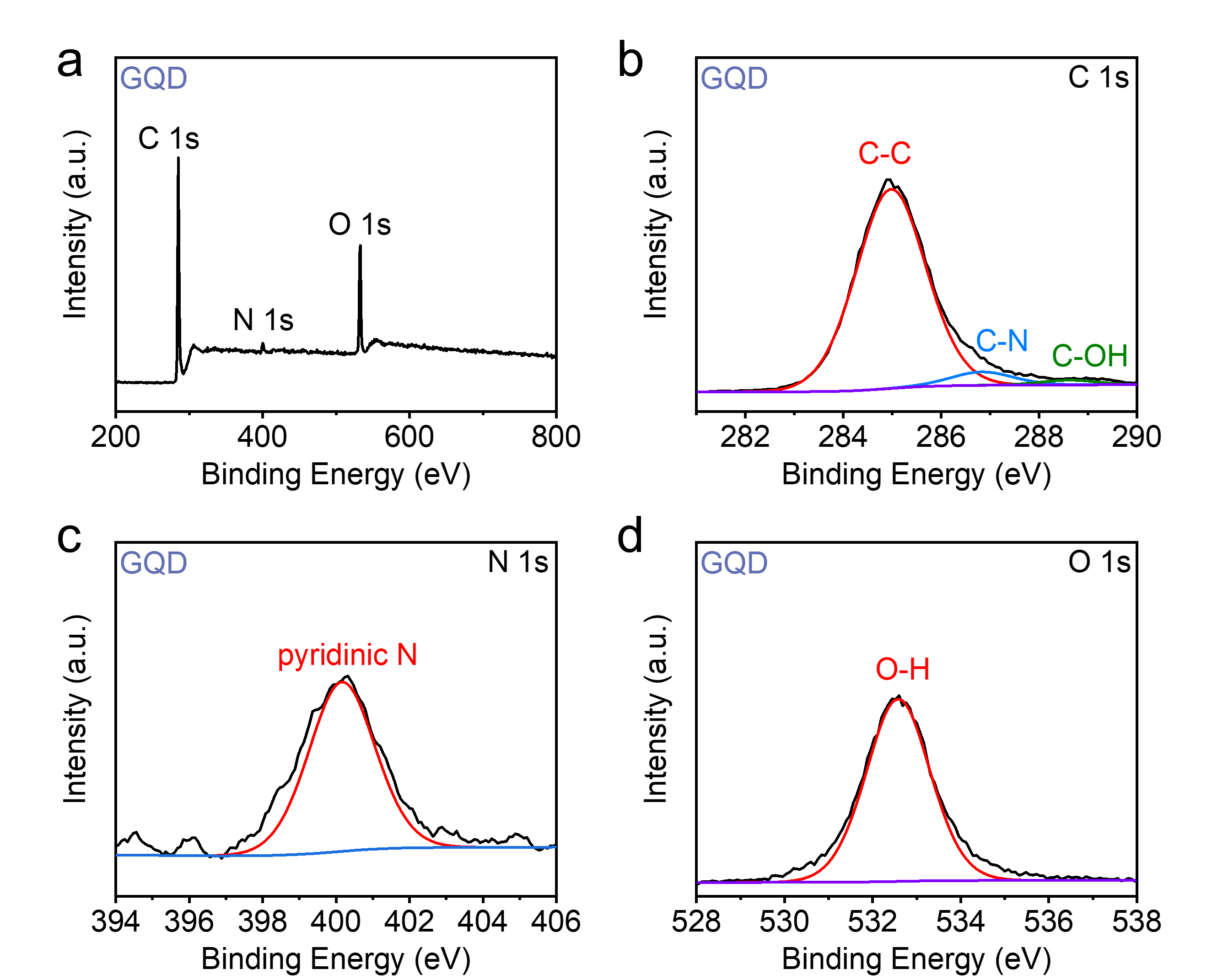


**Figure S3.** Survey XPS spectra (a), high-resolution C 1s (b), N 1s (c), and O 1s (d) spectra of GQDs.


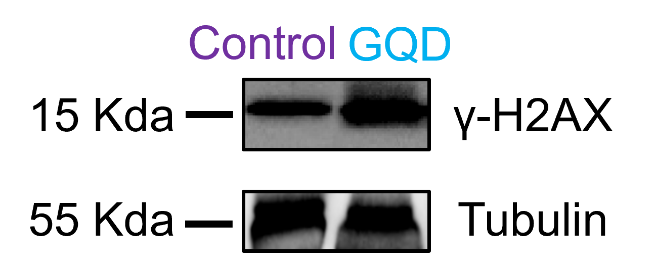


**Figure S4.** Evaluation of the expression levels of γ-H2AX in 4T1 cells after treating with GQD. Tubulin was used as a loading control.


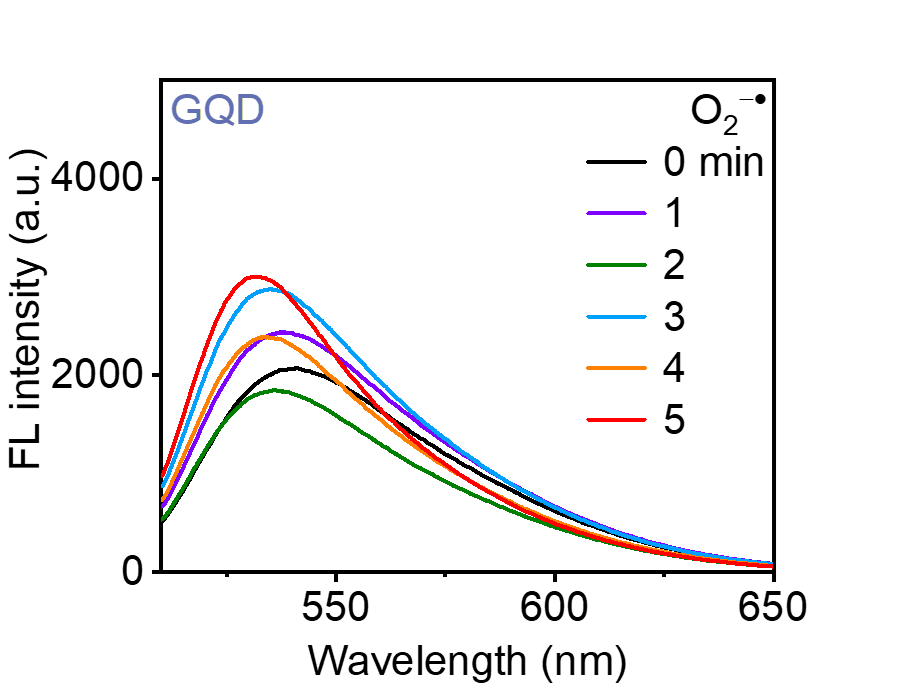


**Figure S5.** Evaluation of the generation of O_2_ˉ^•^ in the presence of GQDs under US irradiation.


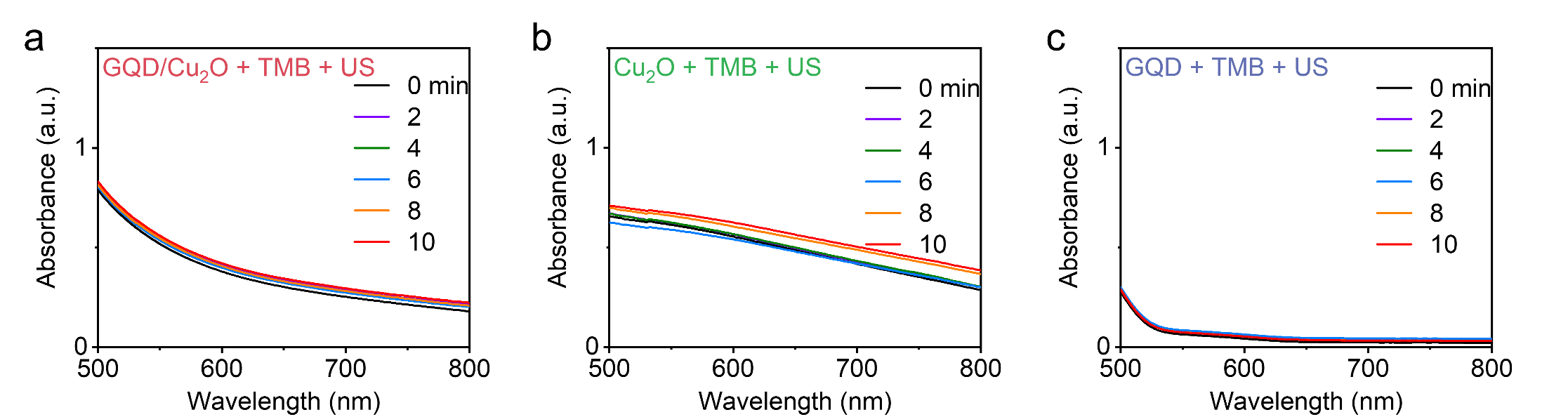


**Figure S6.** Measurements of •OH generation performance of GQD/Cu_2_O, Cu_2_O, and GQD in the presence of US irradiation.


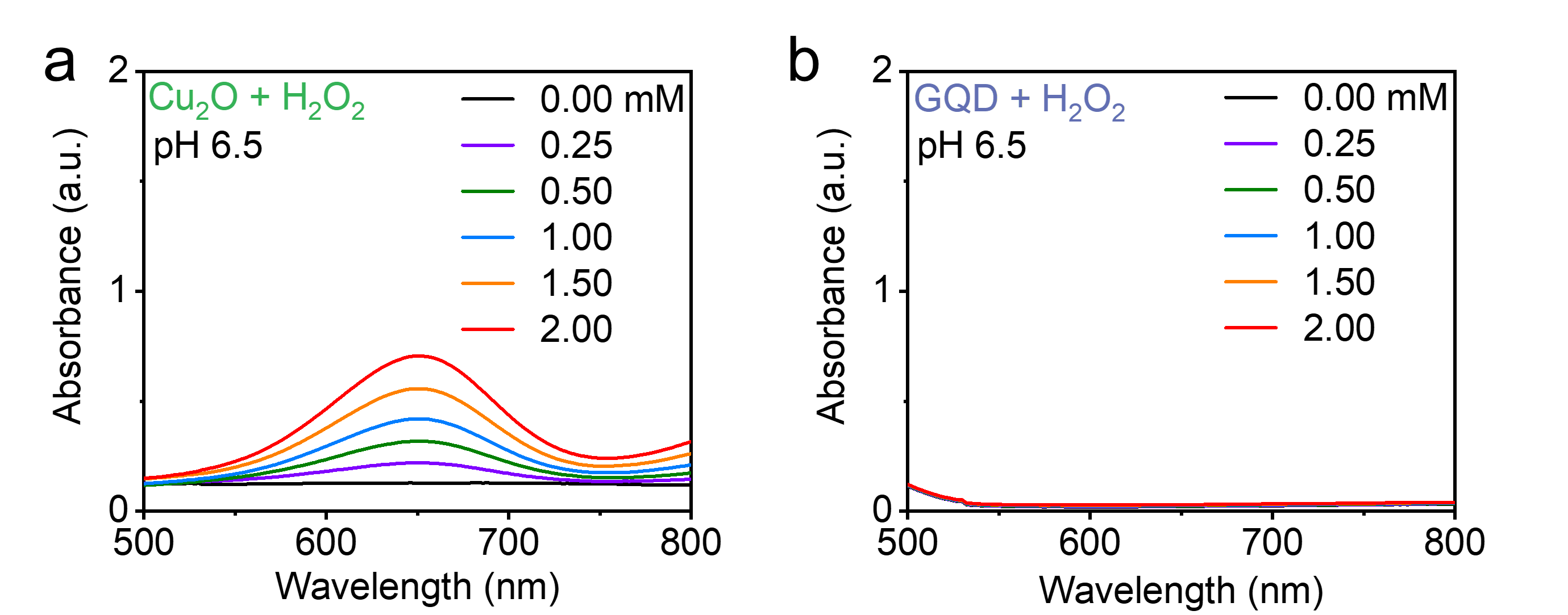


**Figure S7.** Measurements of •OH generation ability of Cu_2_O (a) and GQDs (b) in the presence of H_2_O_2_ with varied concentrations at pH 6.5.


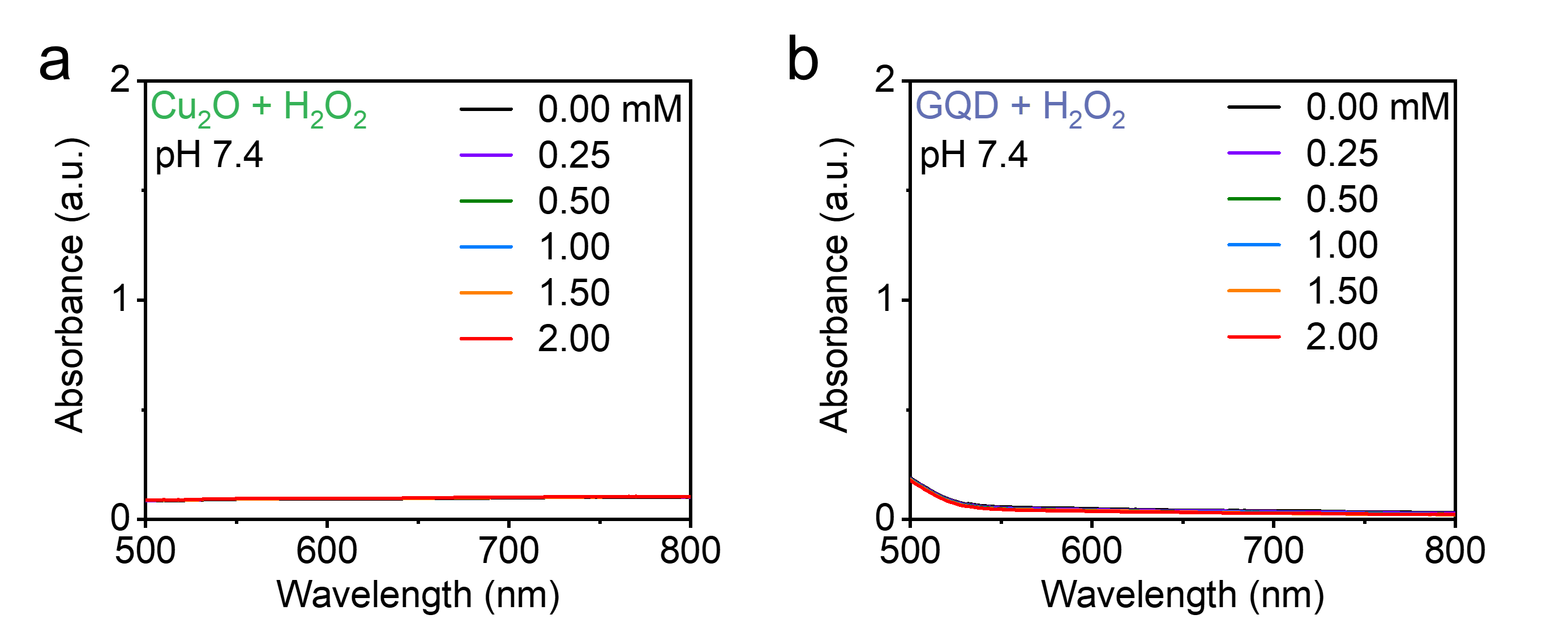


**Figure S8.** Measurements of •OH generation ability of Cu_2_O (a) and GQDs (b) in the presence of H_2_O_2_ with varied concentrations at pH 7.4.


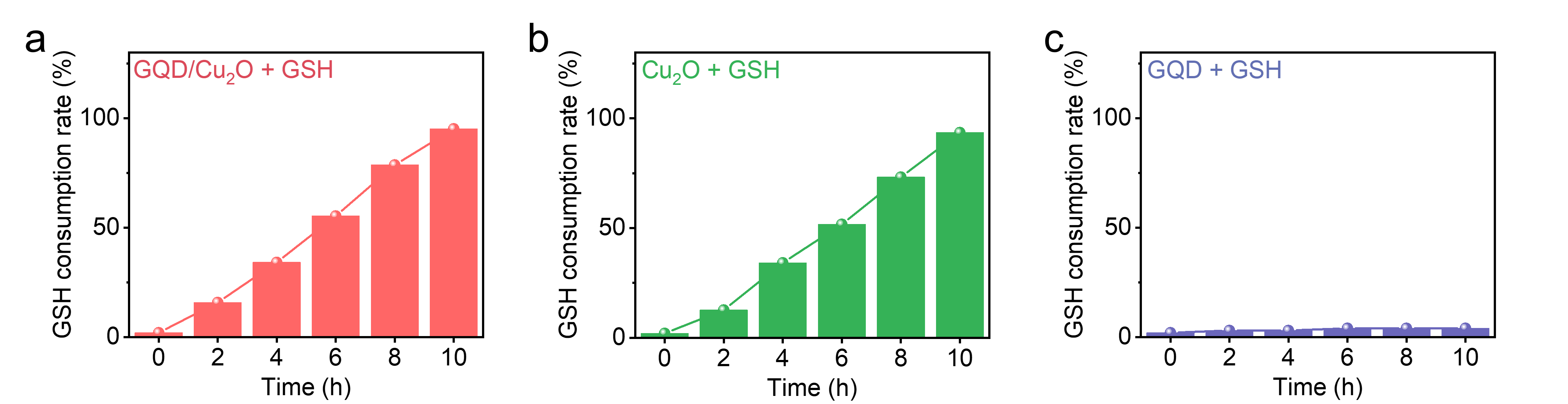


**Figure S9.** Measurements of GSH depletion ability of GQD/Cu_2_O (a), Cu_2_O (b), and GQDs (c).


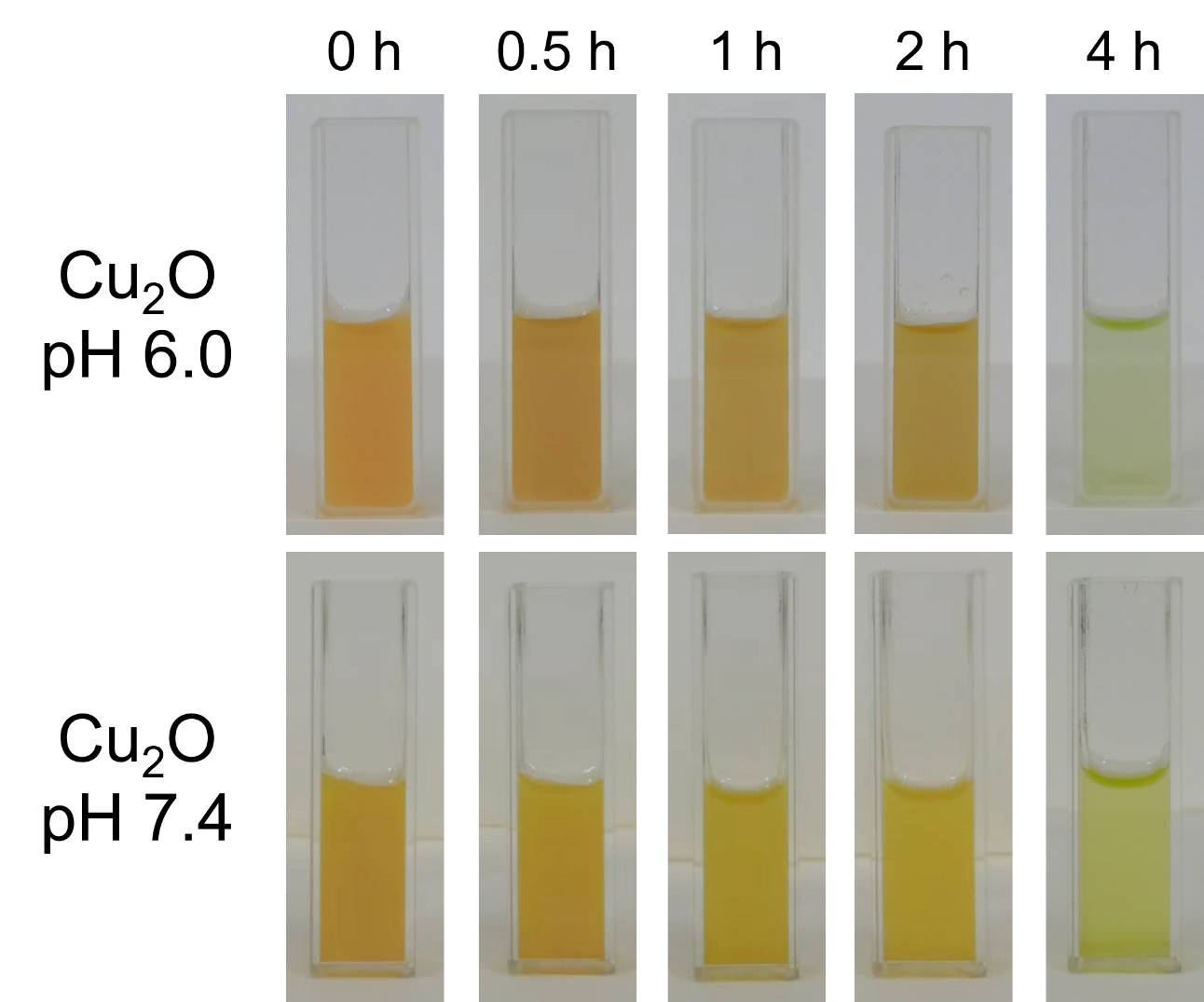


**Figure S10.** Photographs of Cu_2_O solution storing for different times at pH 6.0 and 7.4.


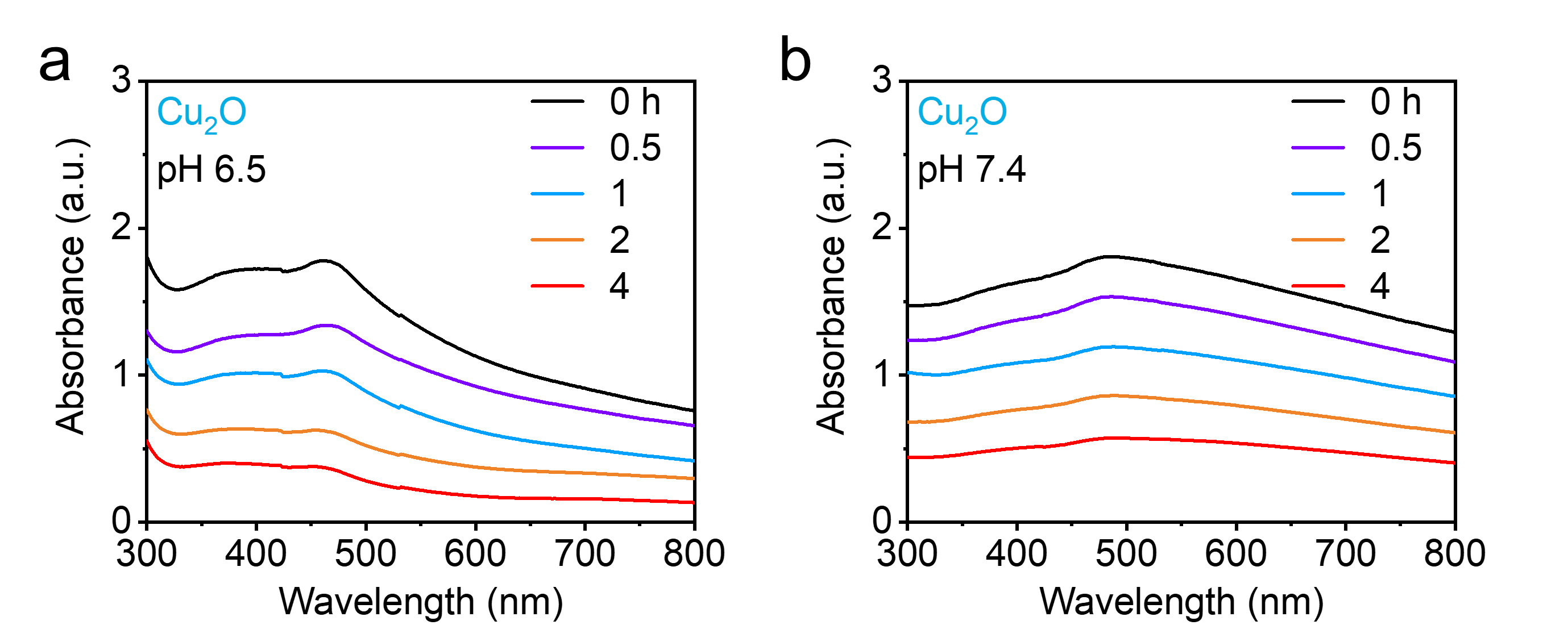


**Figure S11.** Absorption spectra of Cu_2_O after incubation at varied pH for different times.


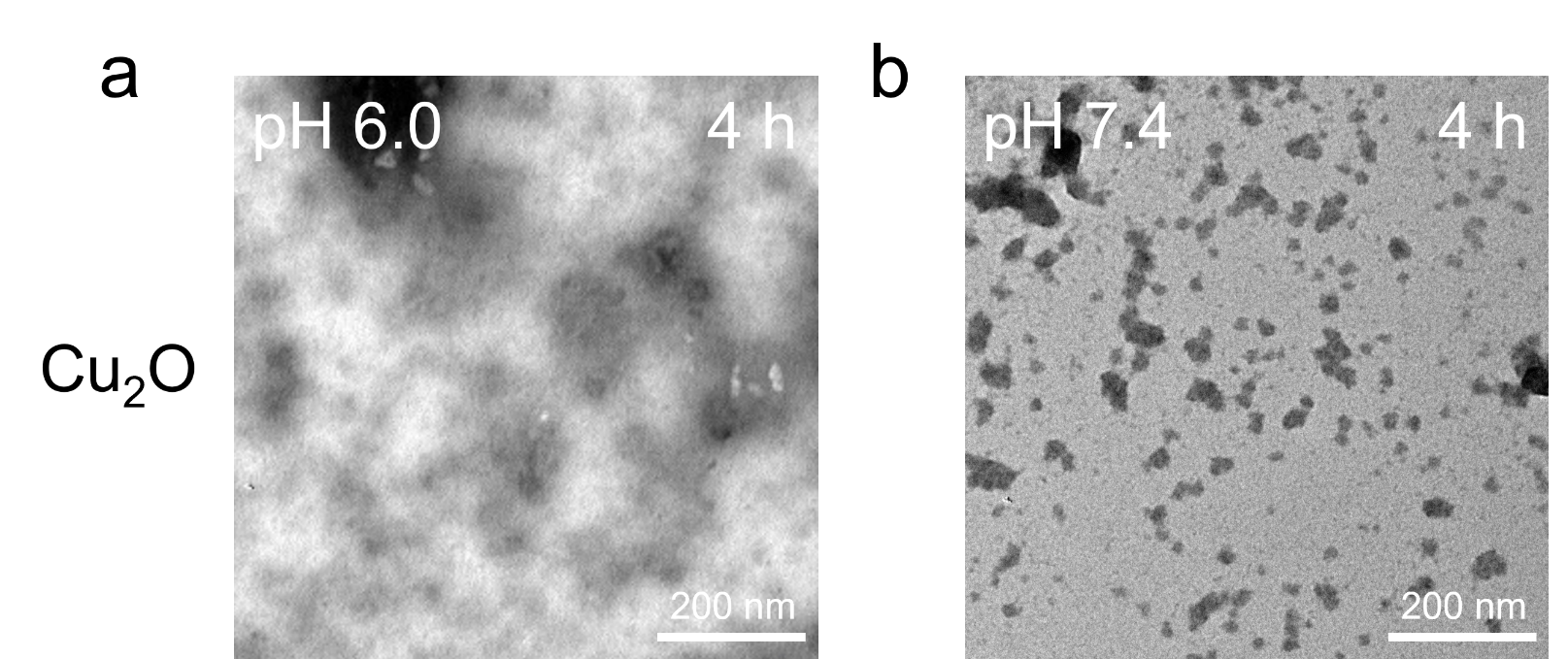


**Figure S12.** TEM iamges of Cu_2_O nanocubes storing for 4 h at pH 6.0 (a) and 7.4 (b).


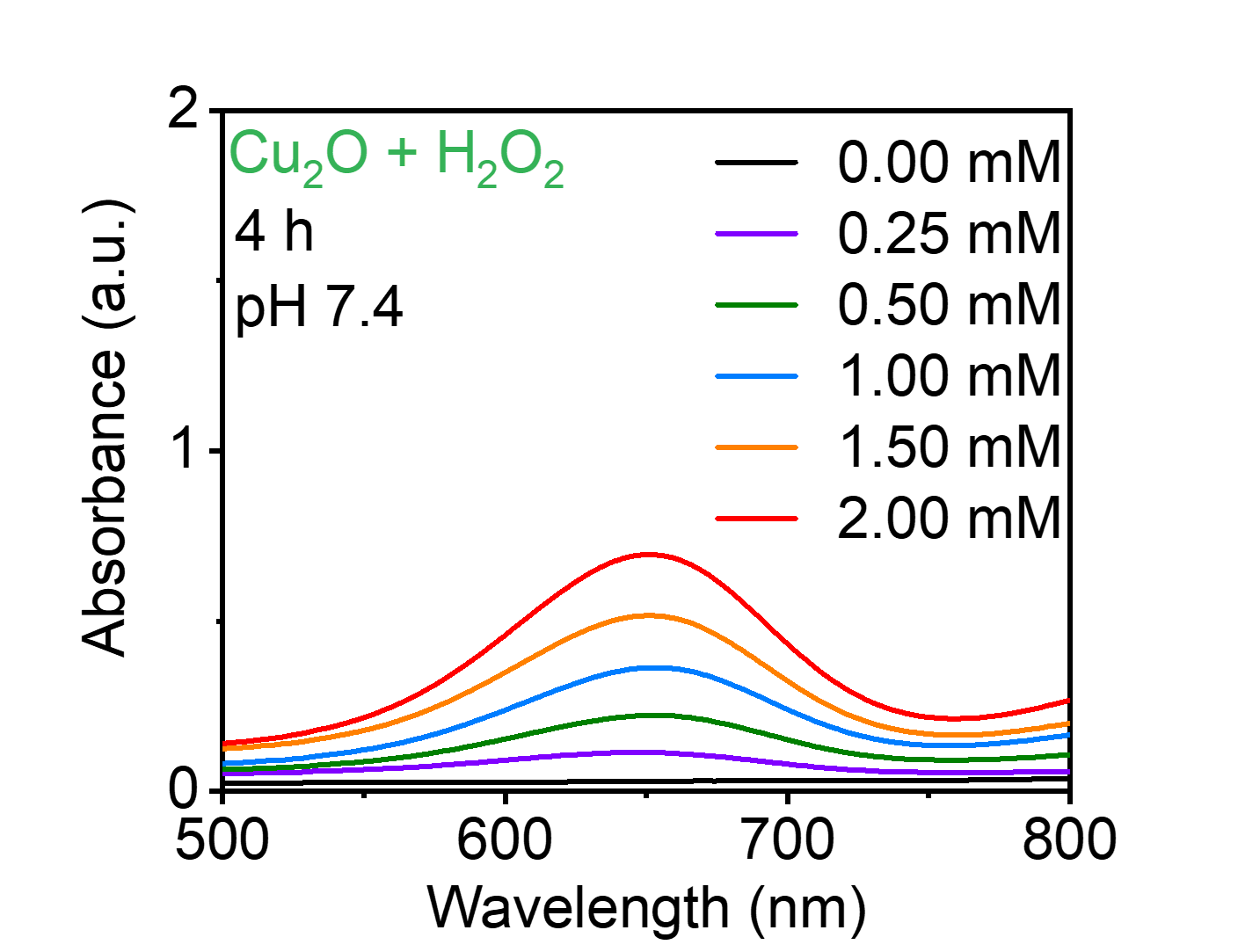


**Figure S13.** Measurements of •OH generation of Cu_2_O after degradation of 4 h at pH 7.4.


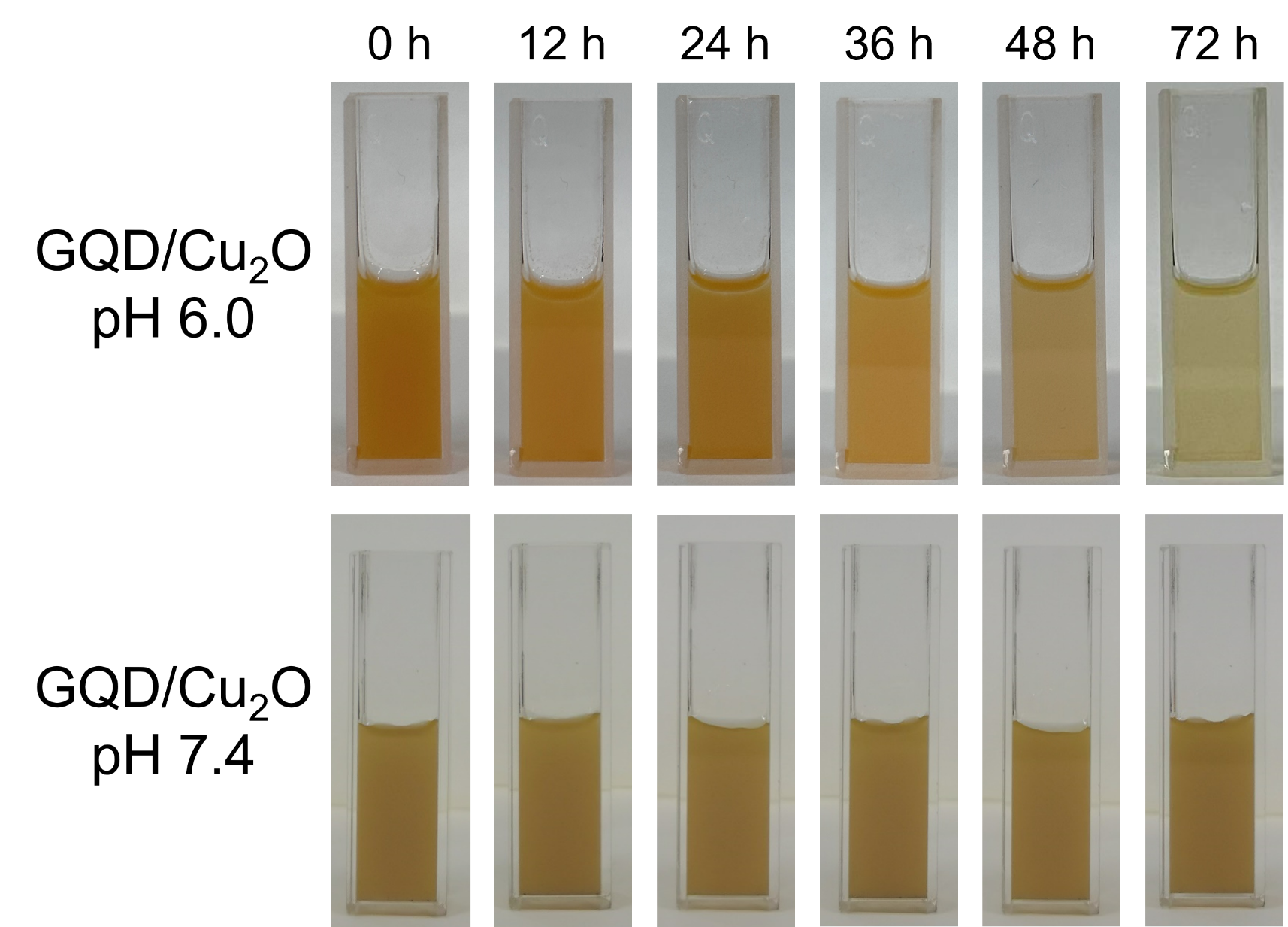


**Figure S14.** Photographs of GQD/Cu_2_O solution storing for different times at pH 6.0 and 7.4.


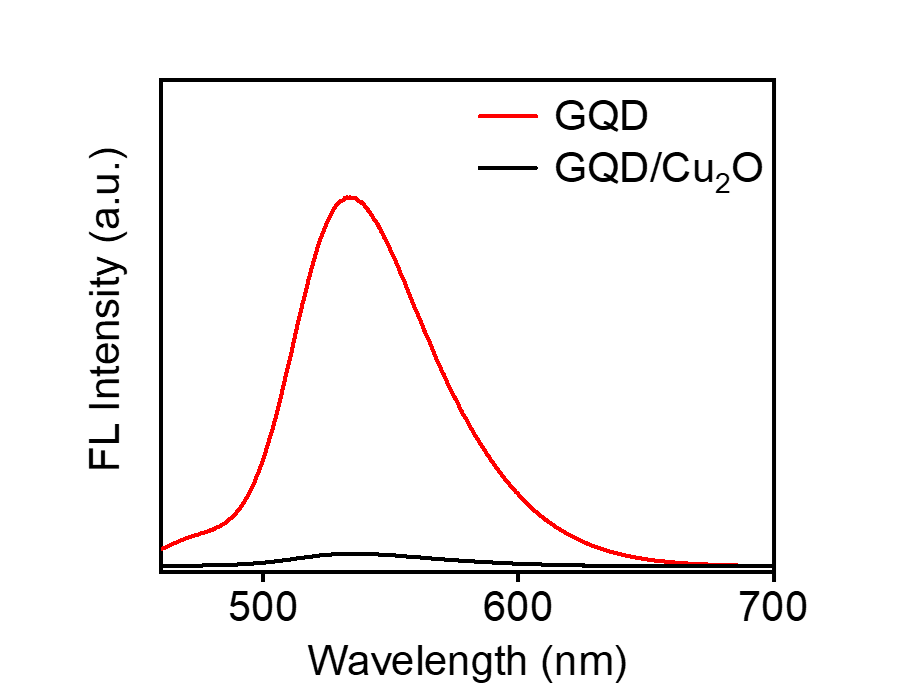


**Figure S15.** Fluorescence spectra of GQDs and GQD/Cu_2_O.


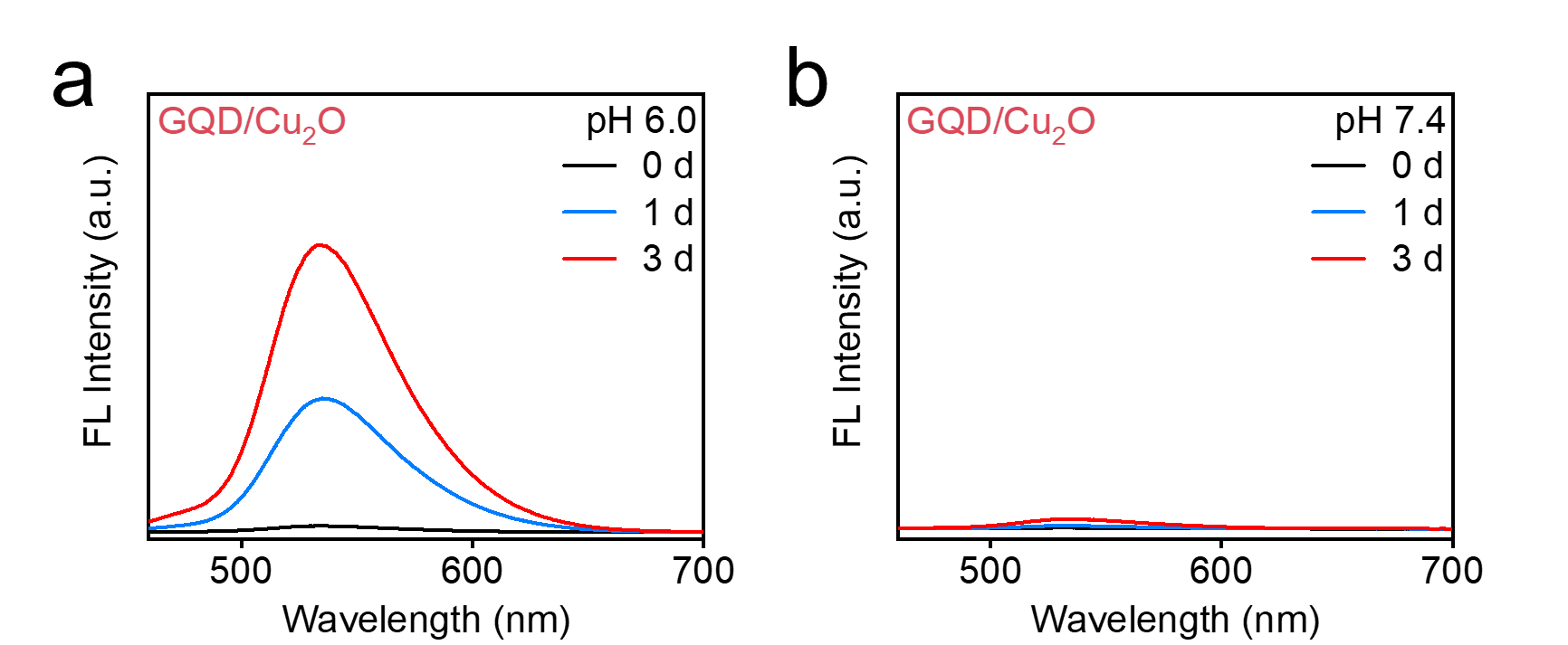


**Figure S16.** Fluorescence spectra of GQD/Cu_2_O storing for different times at pH 6.0 (a) and 7.4 (b).


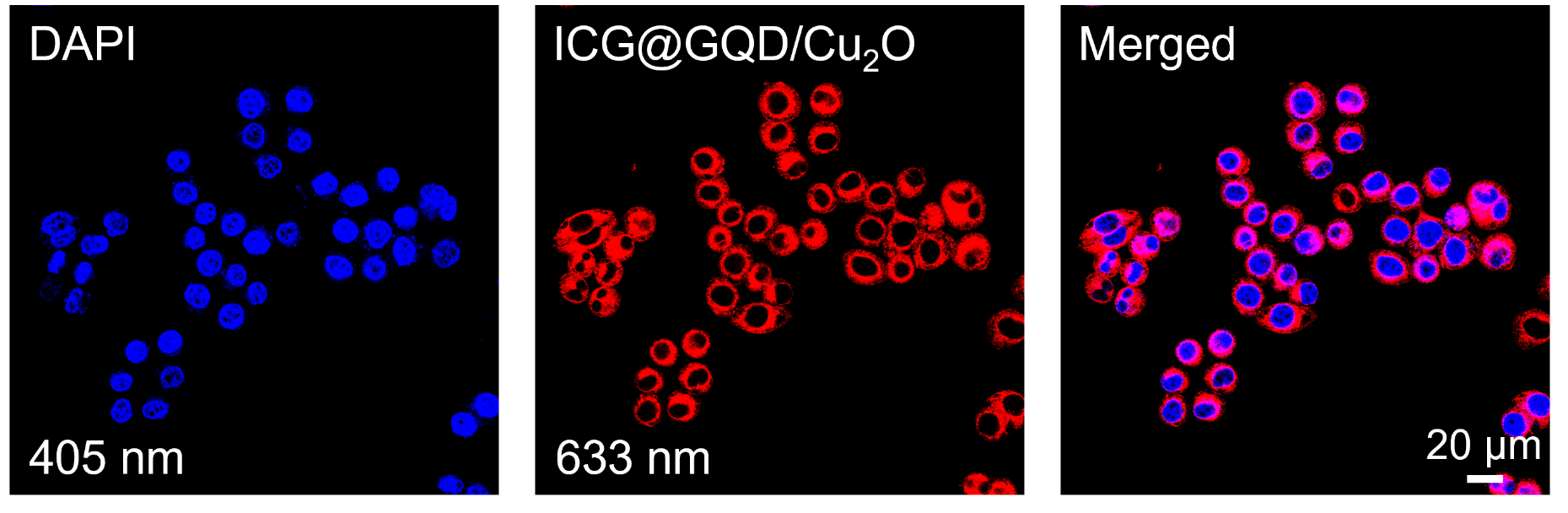


**Figure S17.** Cellular uptake of ICG labeled GQD/Cu_2_O in 4T1 cells.


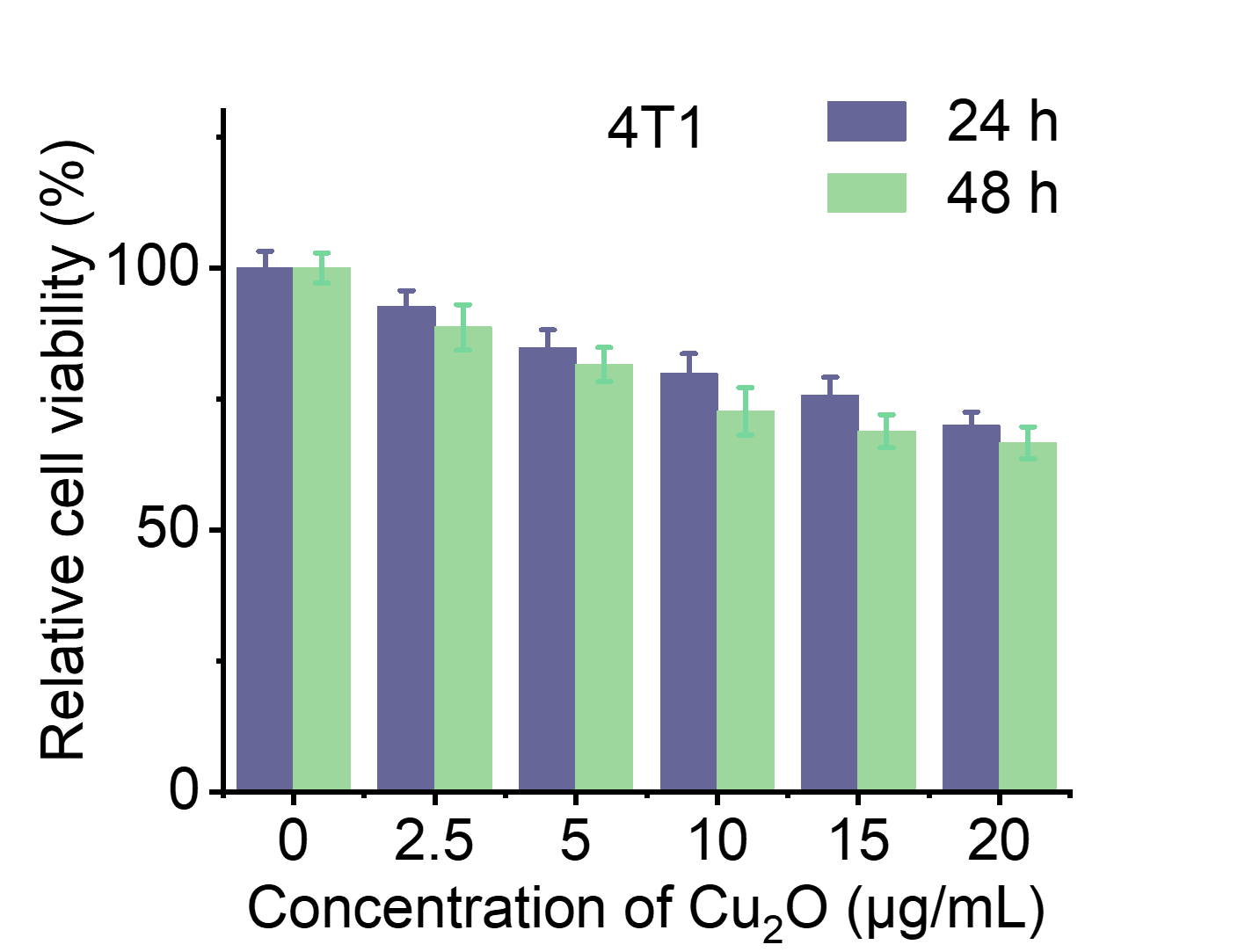


**Figure S18.** Cell viability of 4T1 cells incubated with Cu_2_O. Data are presented as the mean ± SD. (n = 5).


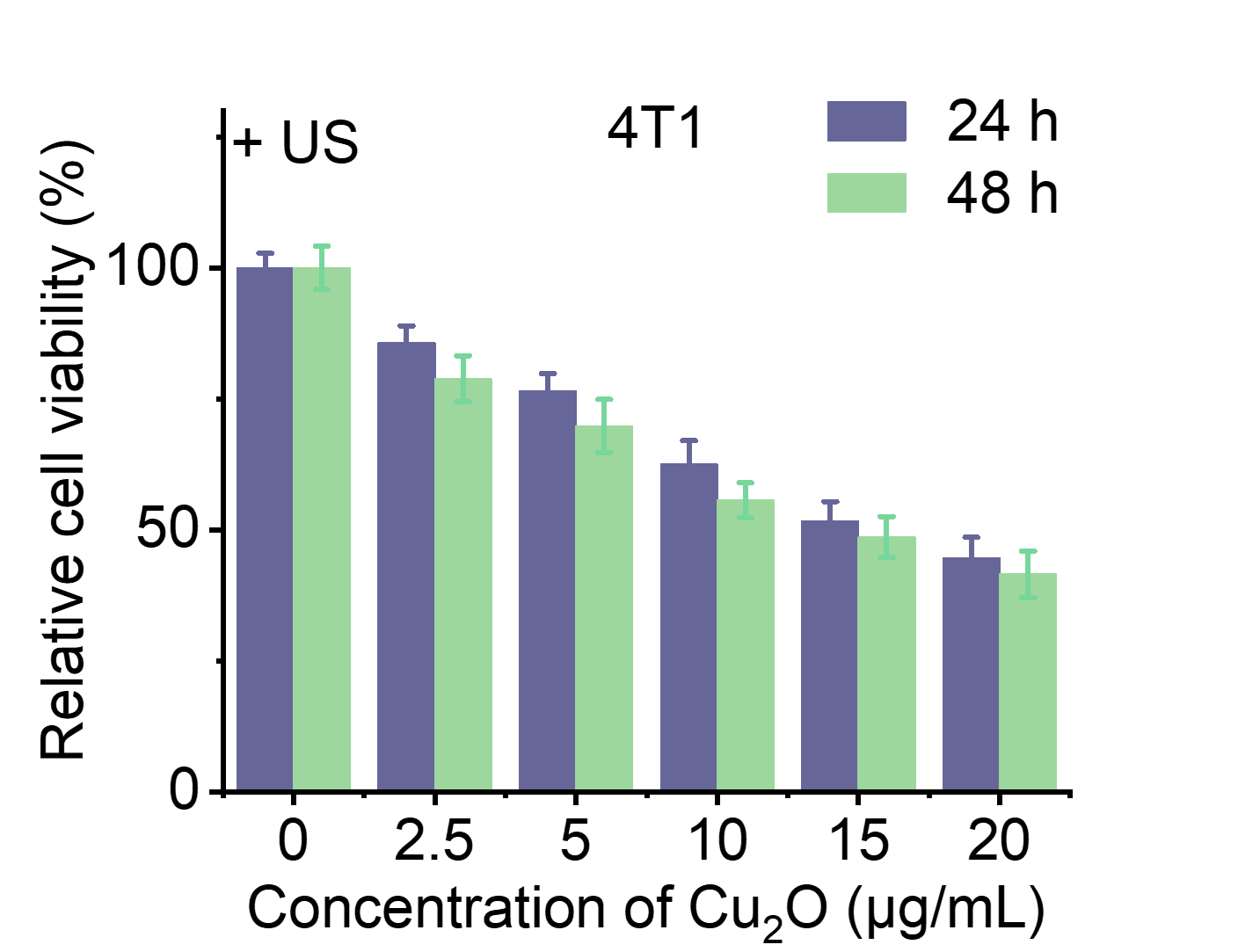


**Figure S19.** Cell viability of 4T1 cells incubated with Cu_2_O under US irradiation. Data are presented as the mean ± SD. (n = 5).


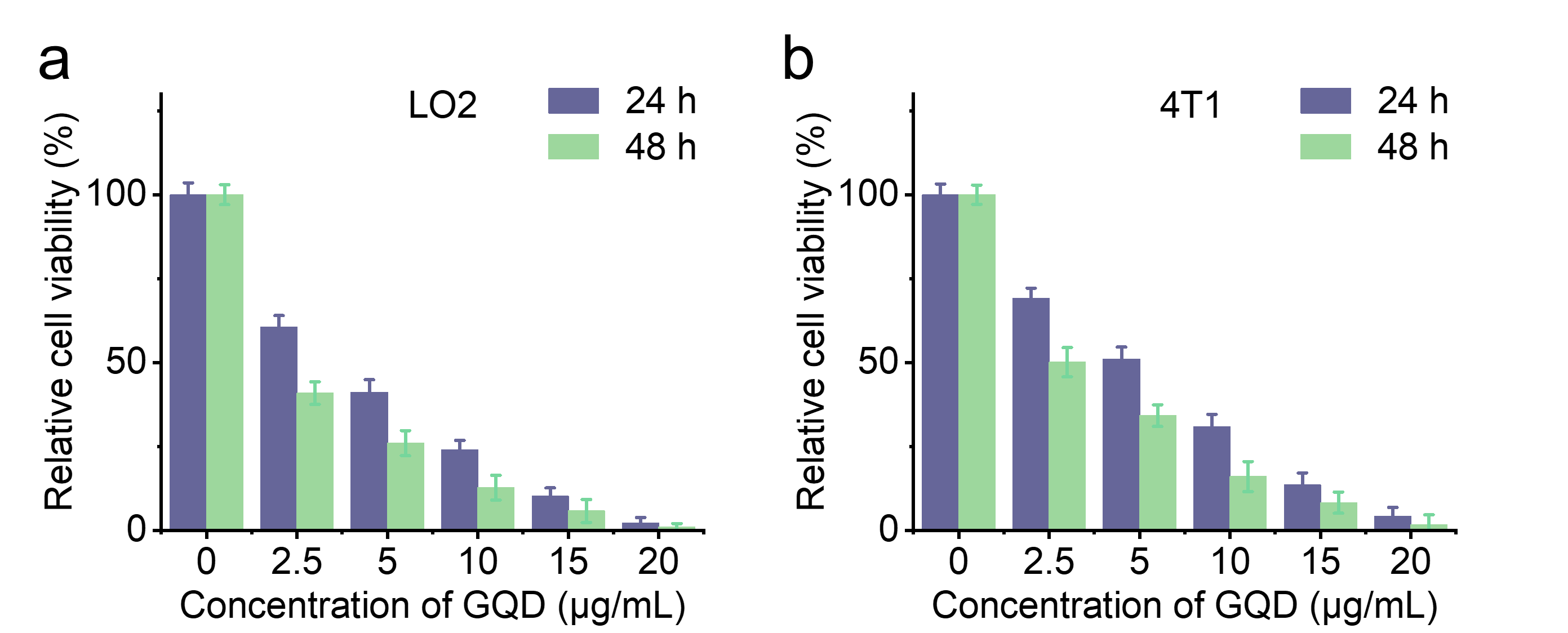


**Figure S20.** Cell viability of LO2 cells incubated with GQD (a) and Cu_2_O (b). Data are presented as the mean ± SD. (n = 5).


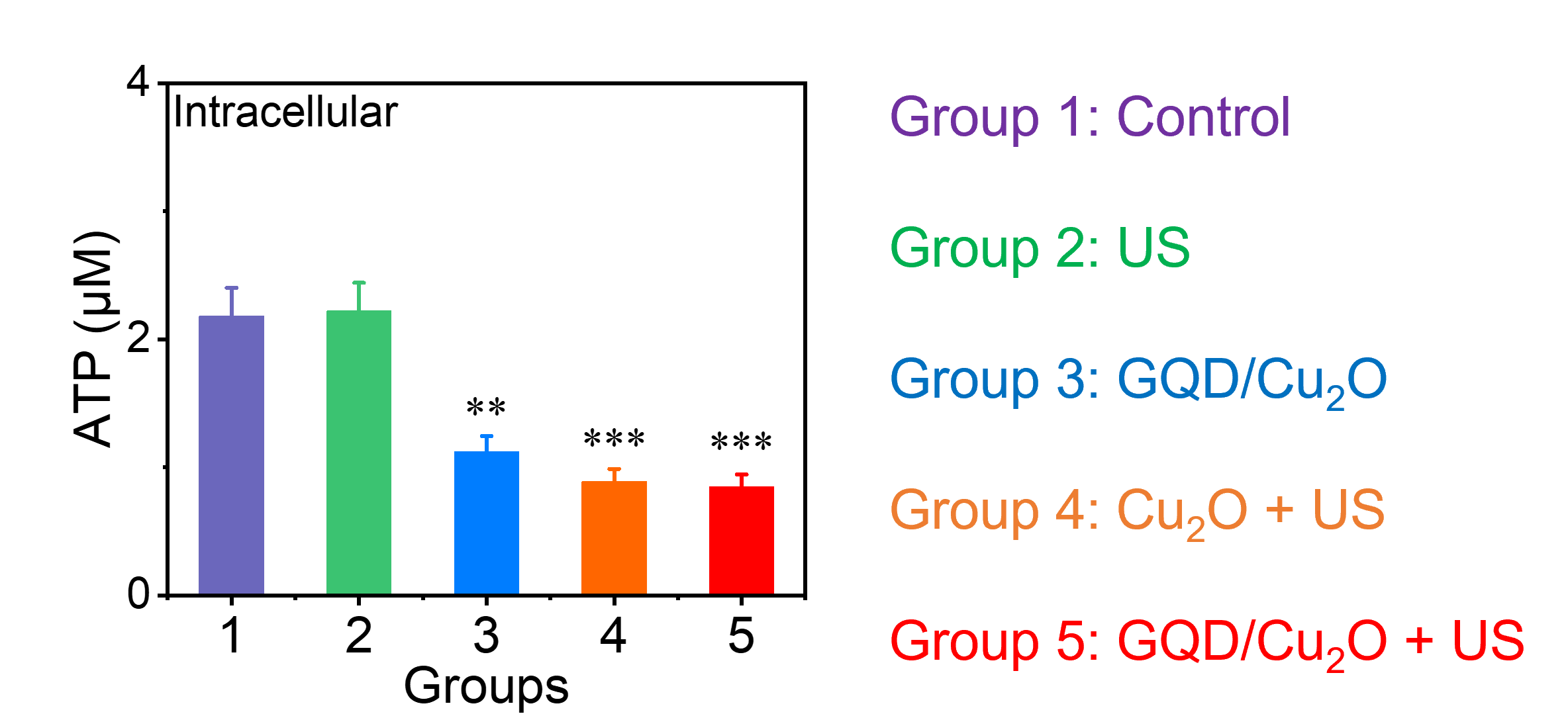


**Figure S21.** Detection of the intracellular ATP level in 4T1 cells after different treatments. Statistical significance between the experimental group and the control group is calculated with a two-tailed Student’s t-test. Data are presented as the mean ± SD. (n = 3). **p < 0.01 and ***p < 0.001.


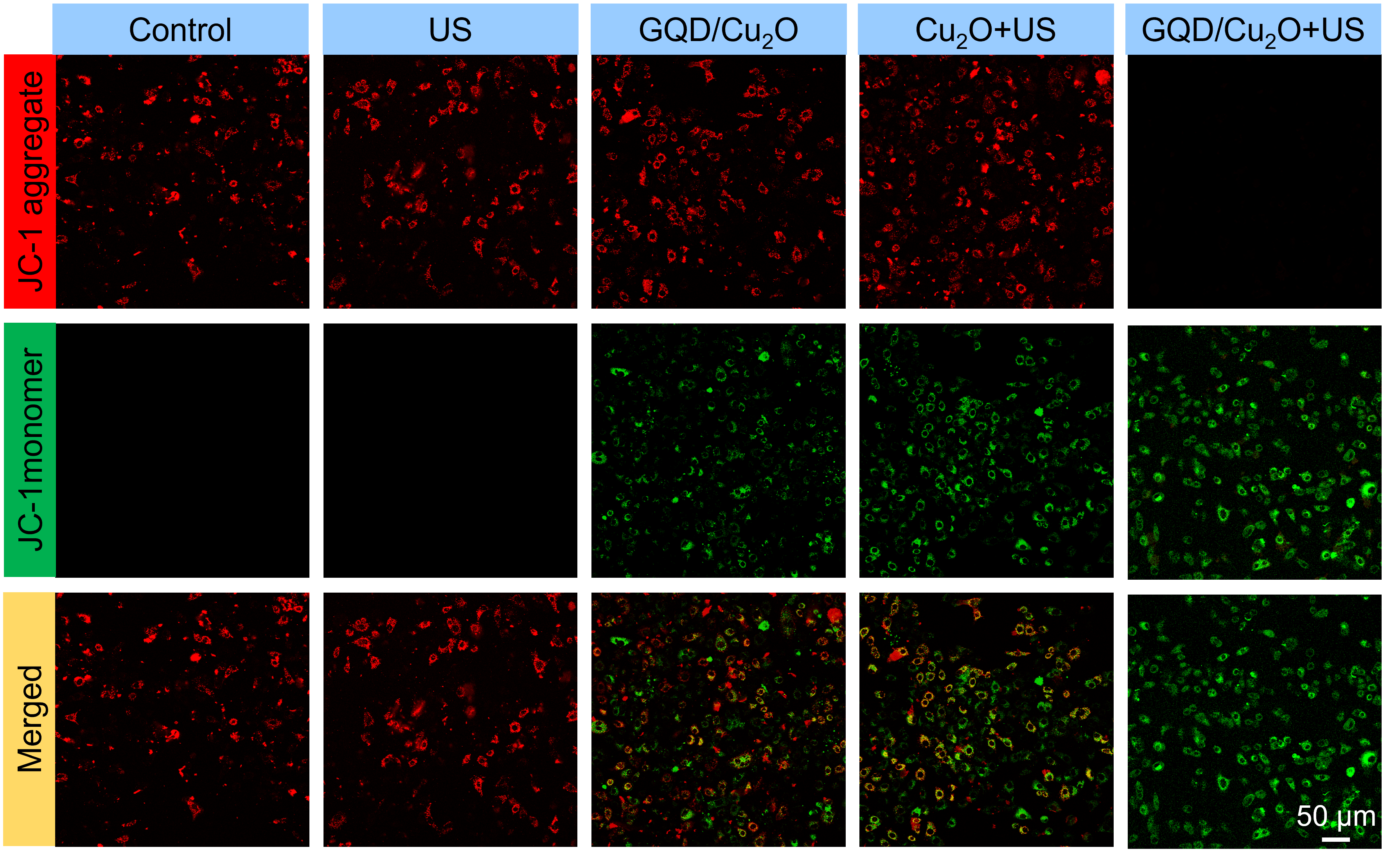


**Figure S22.** JC-1 imaging of 4T1 cells after different treatments.


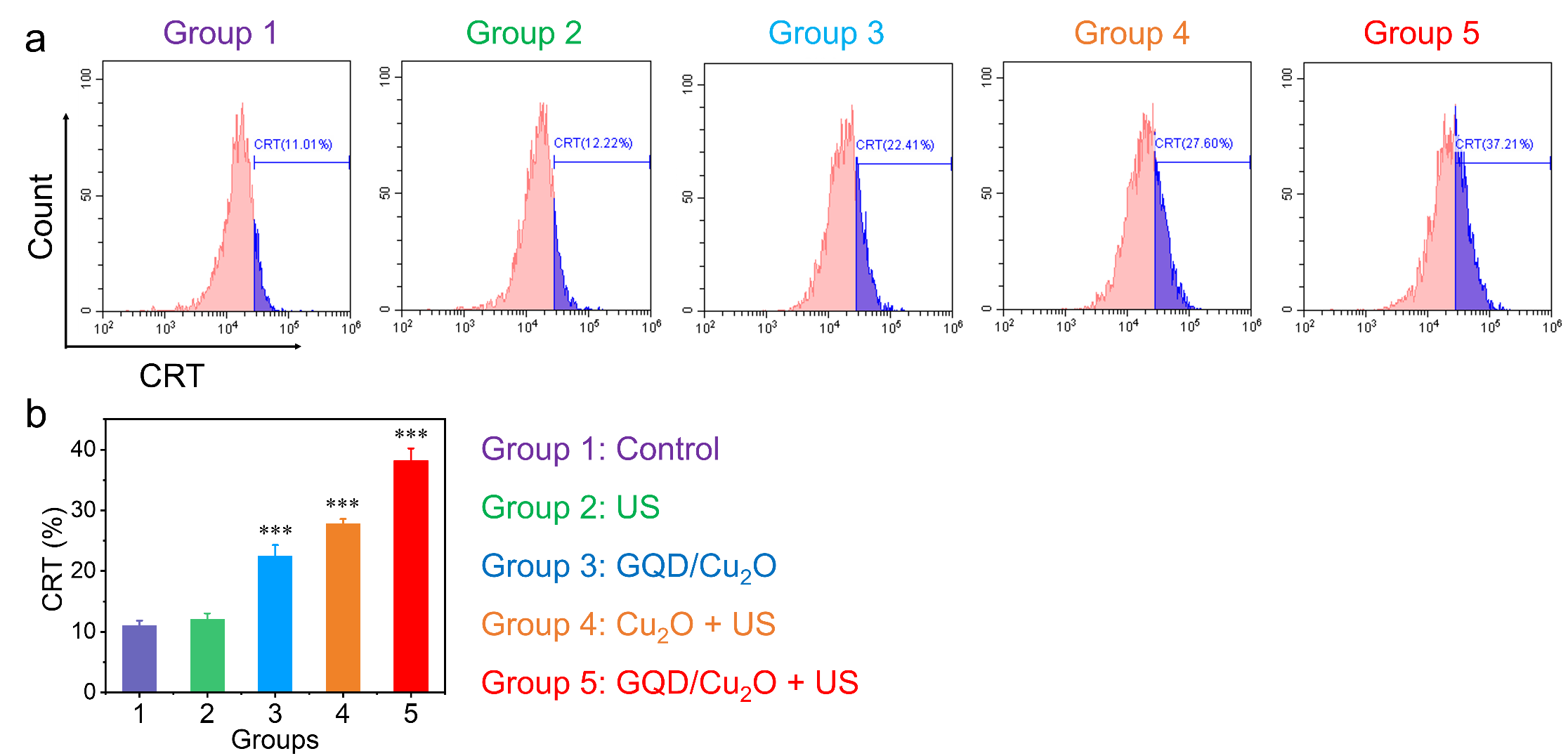


**Figure S23.** (a, b) Flow cytometry analysis and the quantitative results of CRT exposure in 4T1 cells after different treatments.


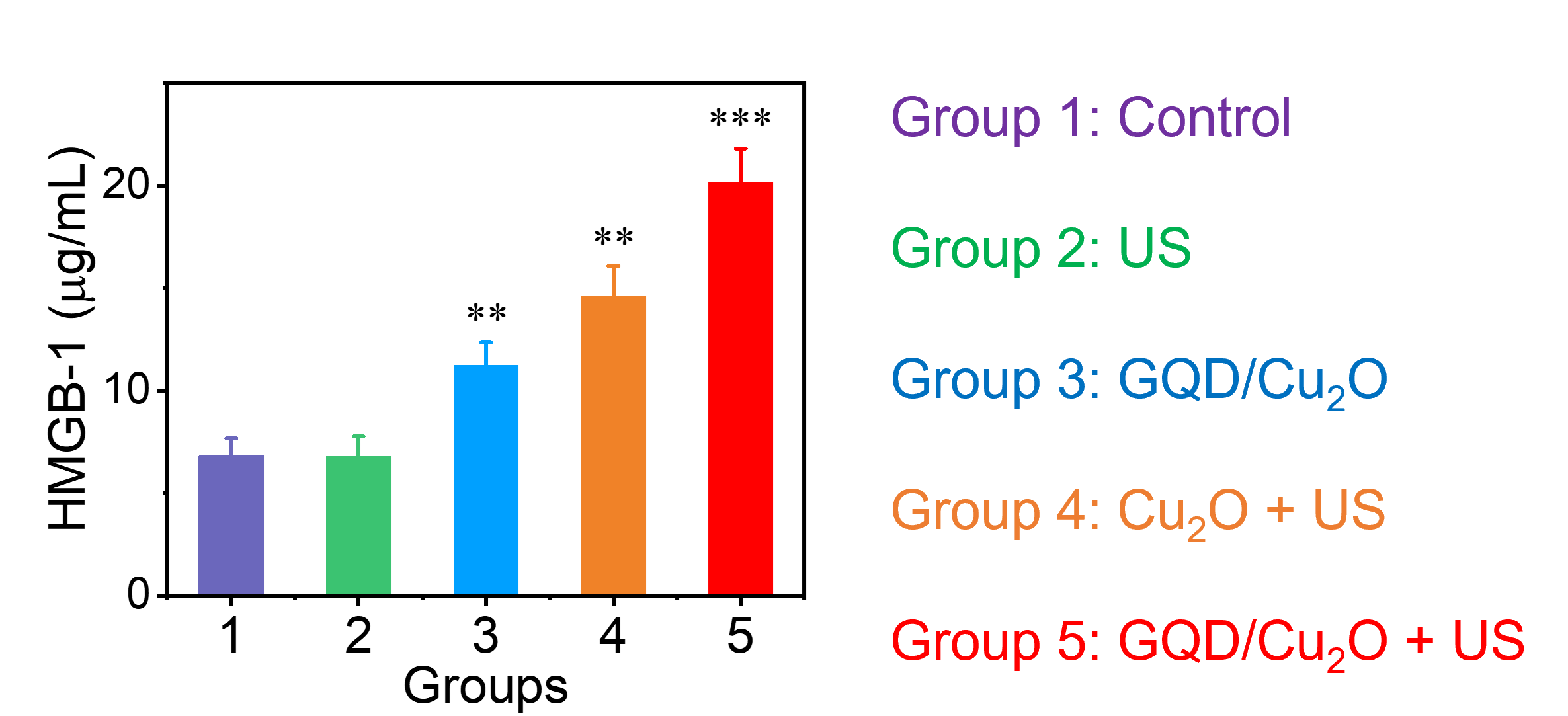


**Figure S24.** Detection of the HMGB1 level in 4T1 cells after different treatments. Statistical significance between the experimental group and the control group is calculated with a two-tailed Student’s t-test. Data are presented as the mean ± SD. (n = 3). **p < 0.01 and ***p < 0.001.


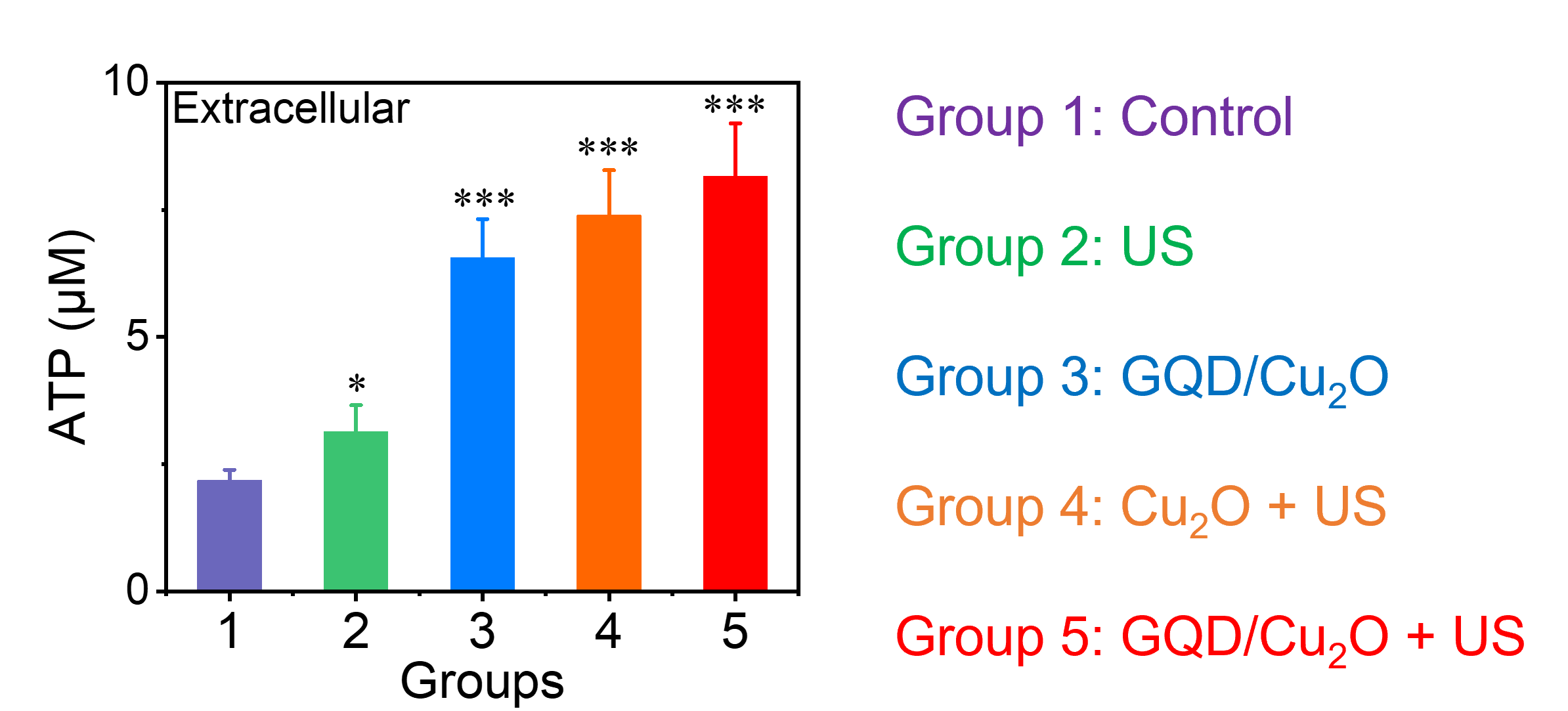


**Figure S25.** Detection of the extracellular ATP level in 4T1 cells after different treatments. Statistical significance between the experimental group and the control group is calculated with a two-tailed Student’s t-test. Data are presented as the mean ± SD. (n = 3). *p < 0.05 and ***p < 0.001.


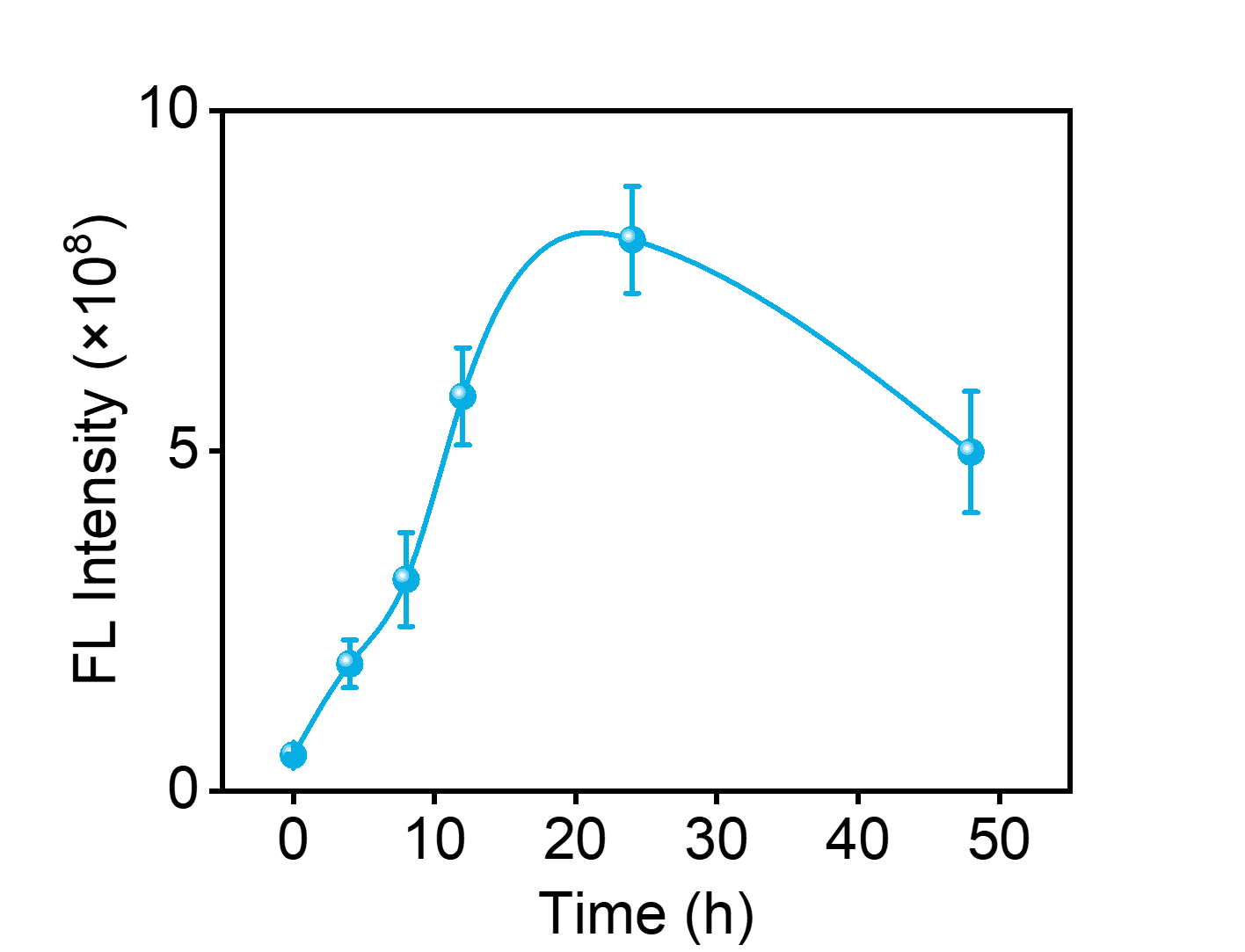


**Figure S26.** The quantitative results of NIR imaging presented in Figure 7b. Data are presented as the mean ± SD. (n = 3).


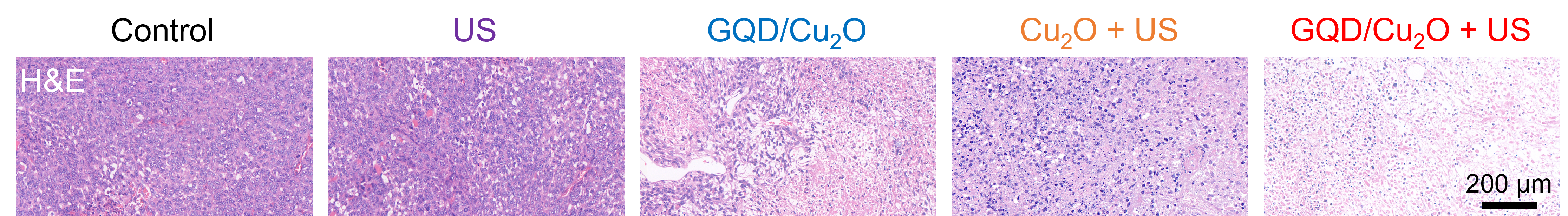


**Figure S27.** H&E staining of distant tumor tissues in mice after different treatments.


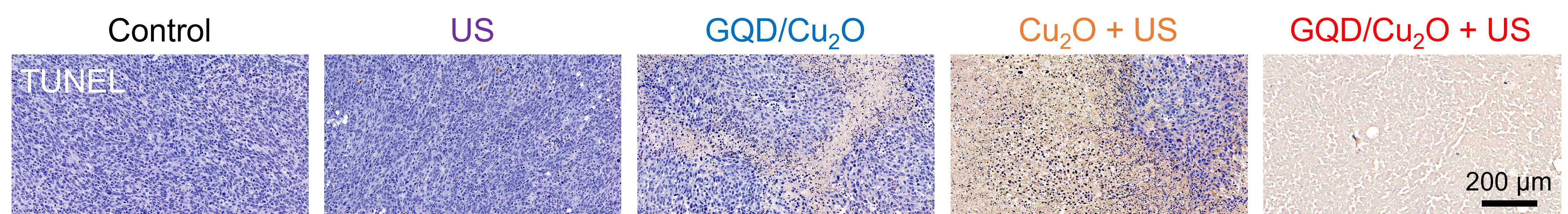


**Figure S28.** TUNEL staining of distant tumor tissues in mice after different treatments.


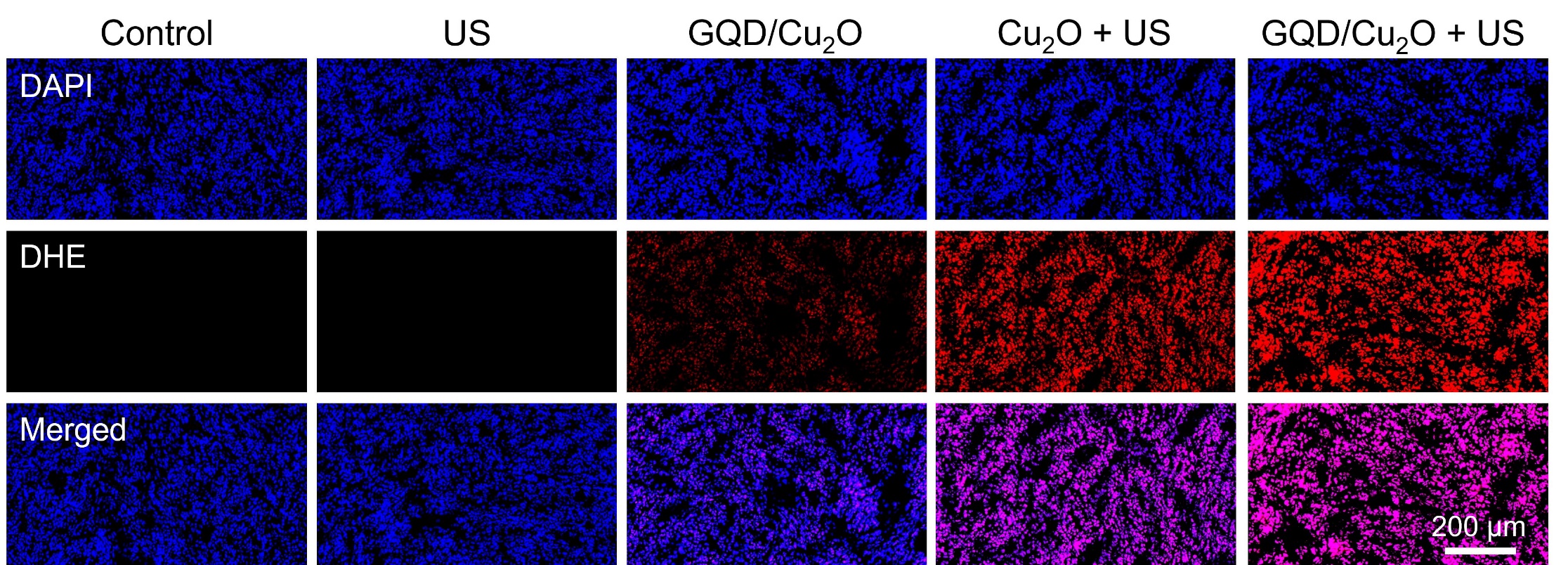


**Figure S29.** ROS levels of tumor tissues of mice after different treatments.


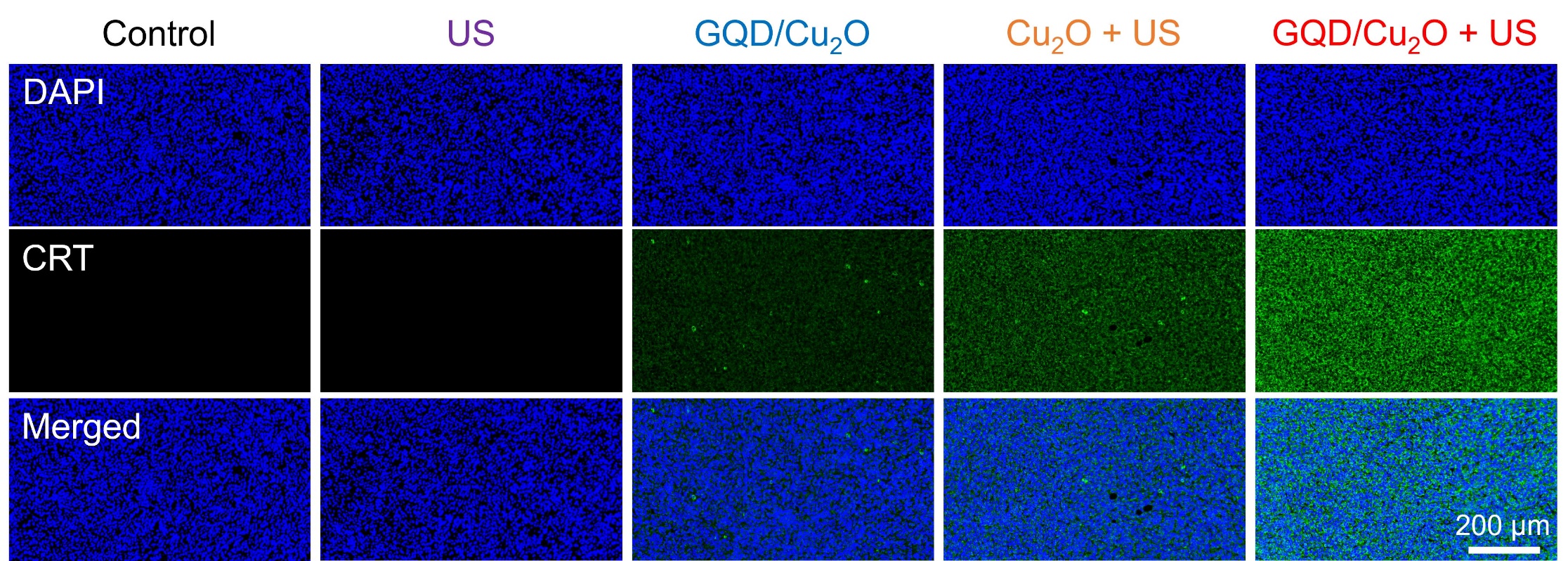


**Figure S30.** CRT levels of tumor tissues of mice after different treatments.


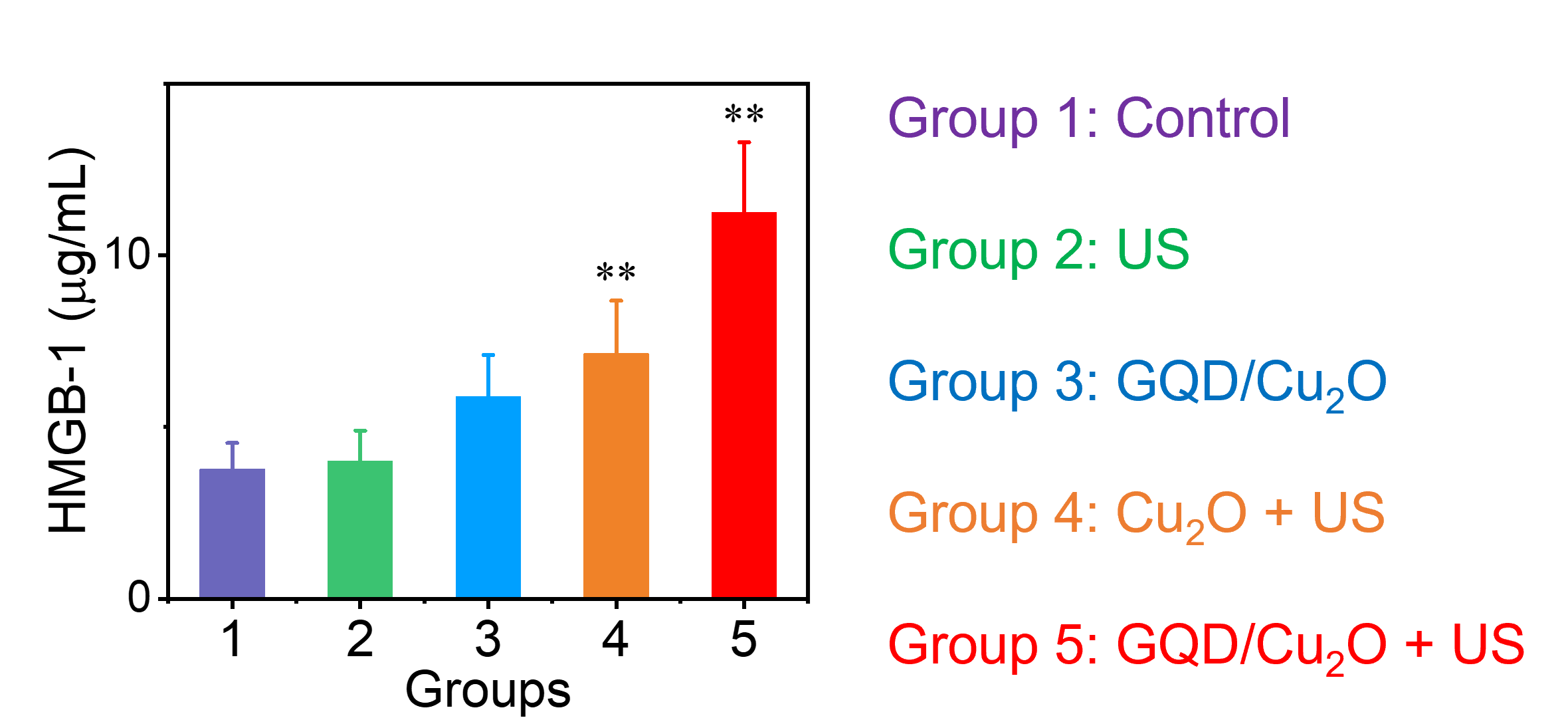


**Figure S31.** HMGB1 levels of tumor tissues of mice after different treatments. Statistical significance between the experimental group and the control group is calculated with a two-tailed Student’s t-test. Data are presented as the mean ± SD. (n = 3). **p < 0.01.


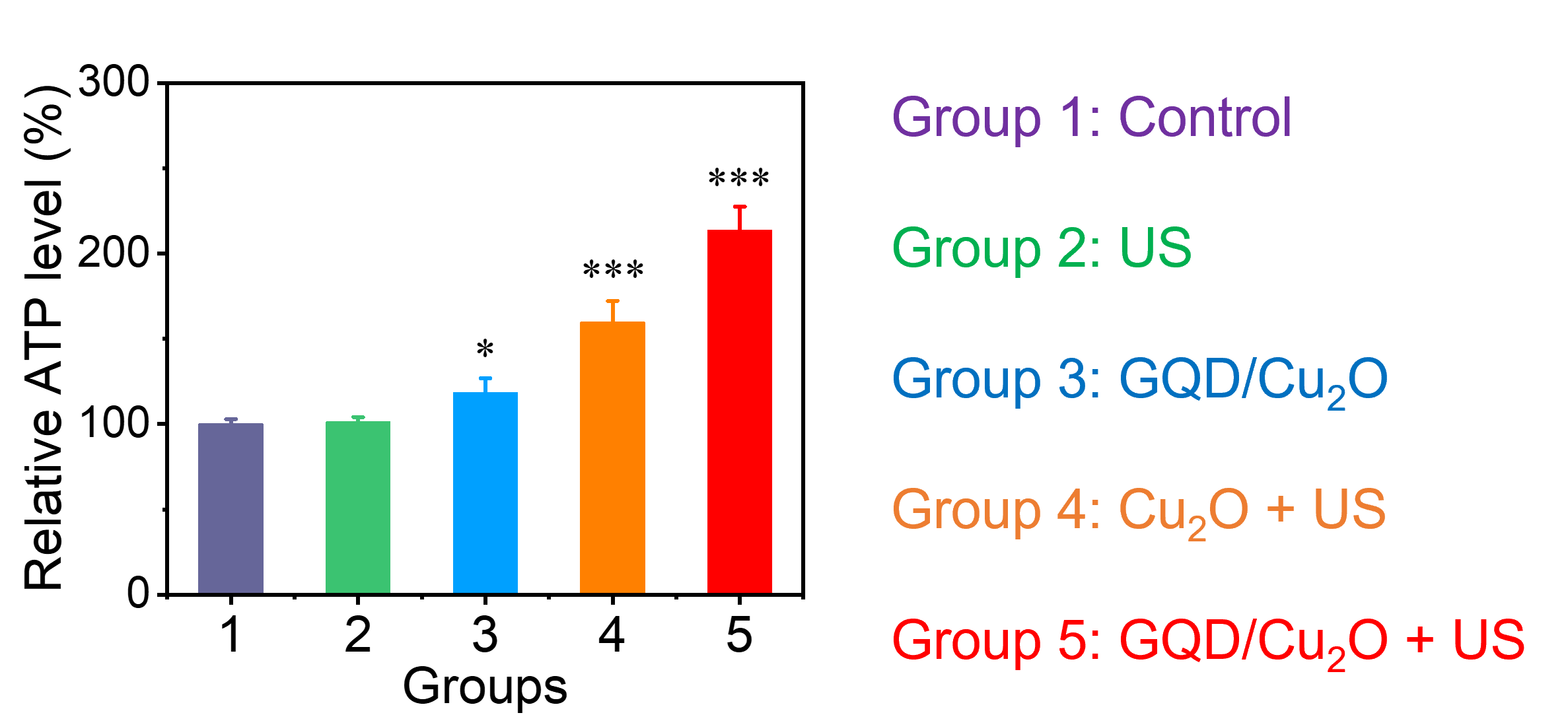


**Figure S32.** ATP levels of tumor tissues of mice after different treatments. Statistical significance between the experimental group and the control group is calculated with a two-tailed Student’s t-test. Data are presented as the mean ± SD. (n = 3). *p < 0.05 and ***p < 0.001.


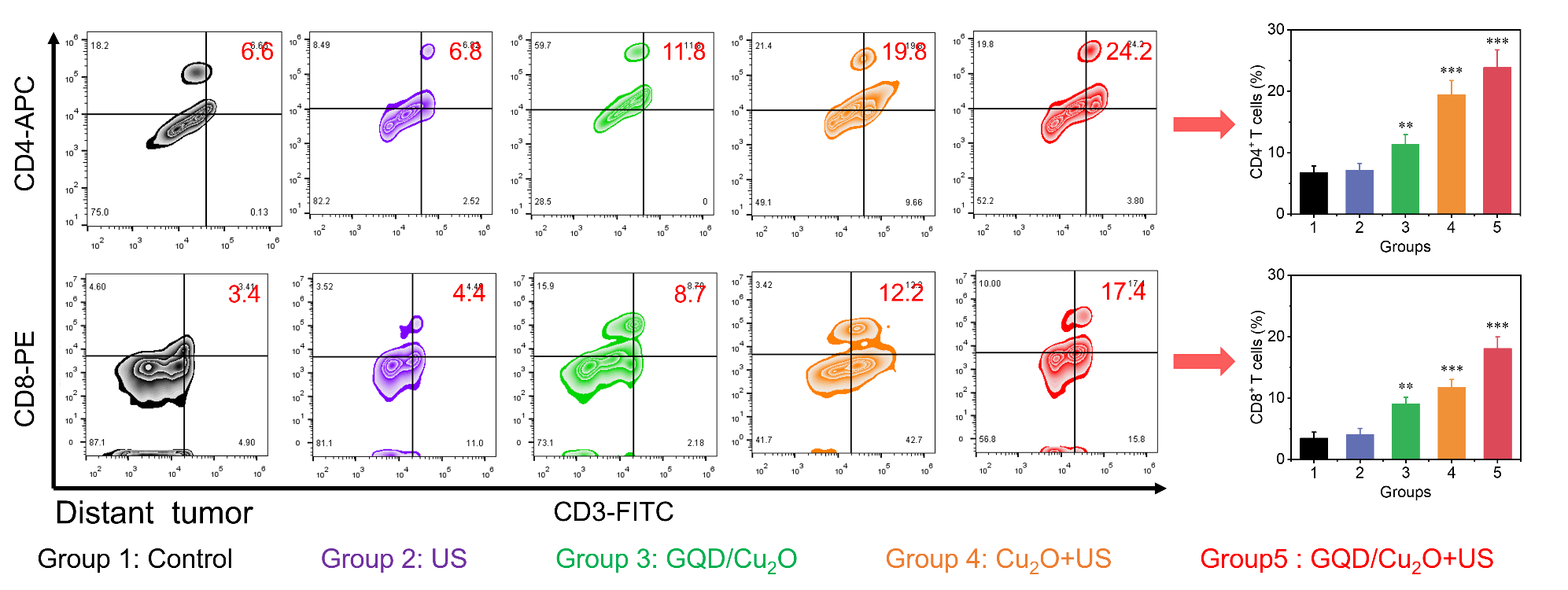


**Figure S33.** Evaluation of the expression of CD4^+^CD8^+^ T cells in the spleen and primary tumors after different treatments. Statistical significance between the experimental group and the control group is calculated with a two-tailed Student’s t-test. Data are presented as the mean ± SD. (n = 3). **p < 0.01 and ***p < 0.001.


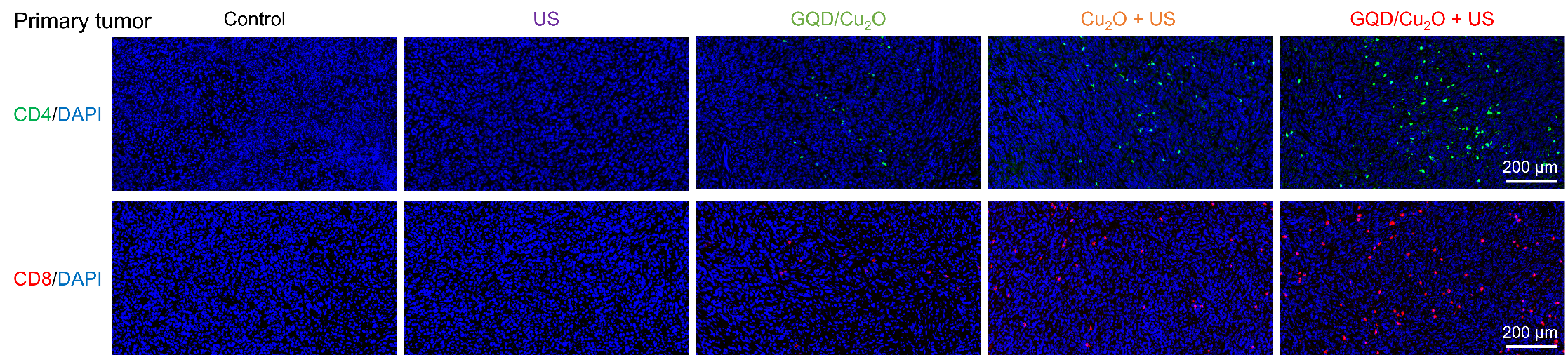


**Figure S34.** Immunofluorescence analysis of CD4^+^ and CD8^+^ T cell infiltration in the primary tumors of mice after different treatments.


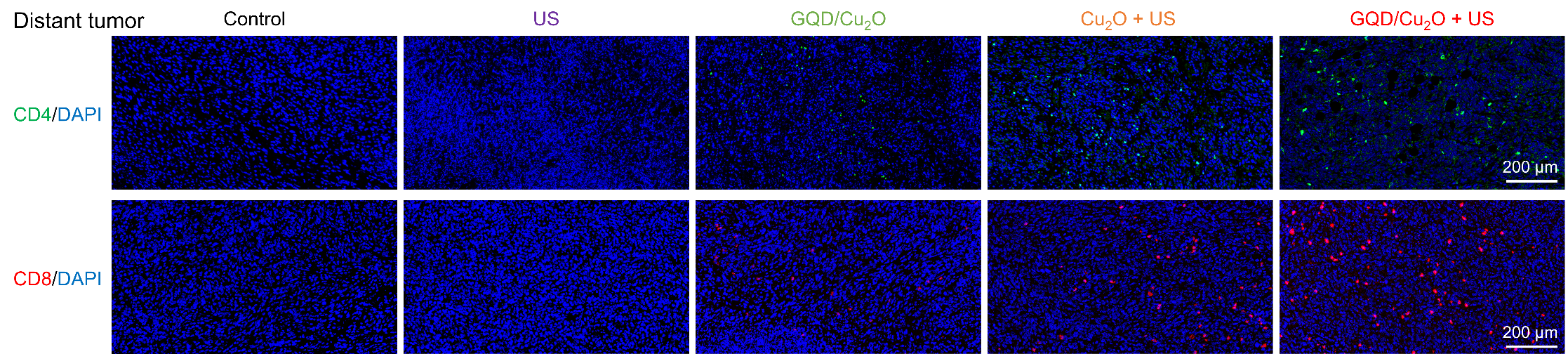


**Figure S35.** Immunofluorescence analysis of CD4^+^ and CD8^+^ T cell infiltration in the distant tumors of mice after different treatments.


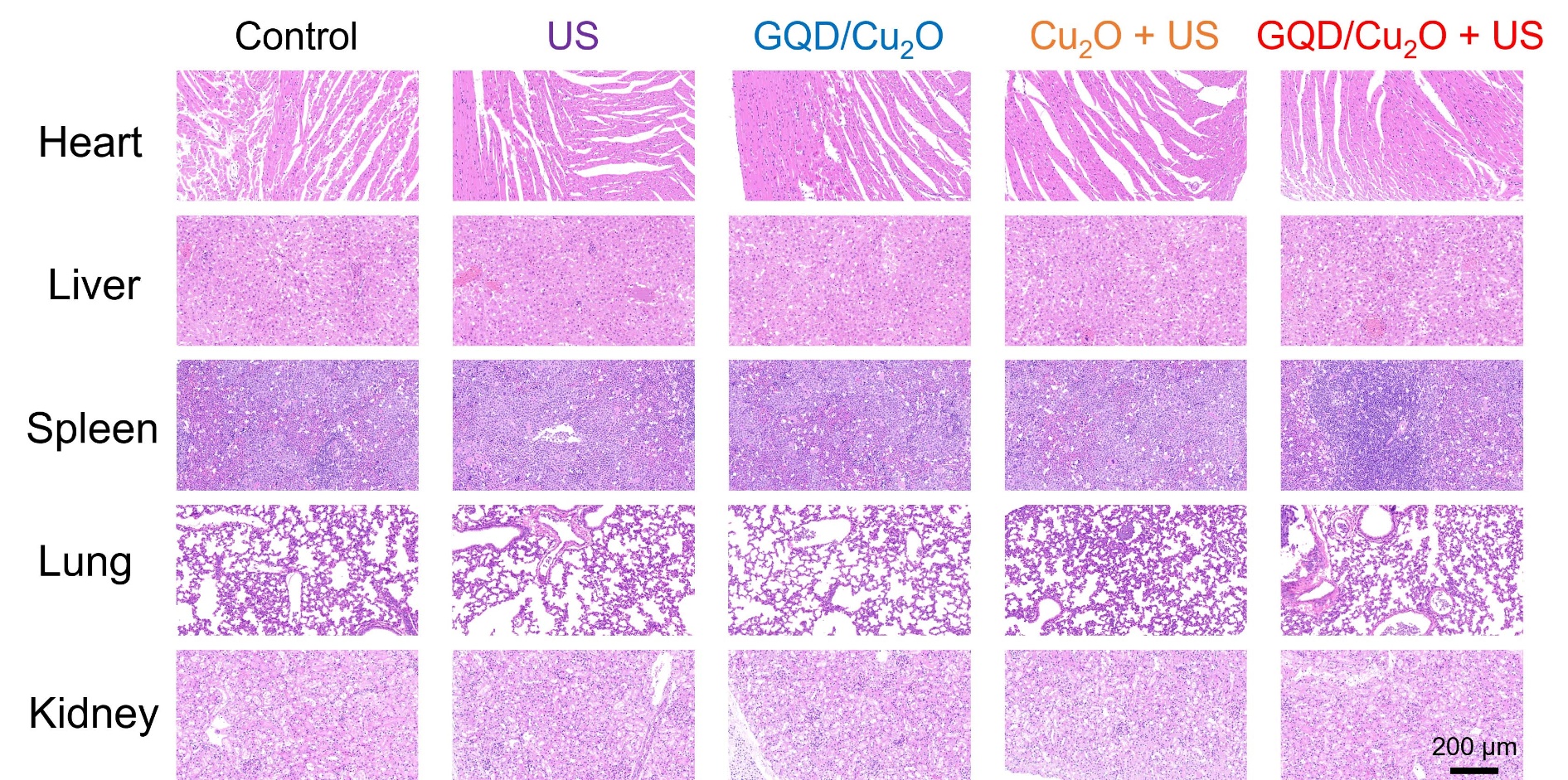


**Figure S36.** H&E-stained images obtained from the major organs (heart, liver, spleen, lung, and kidney) of mice in different treatment groups.


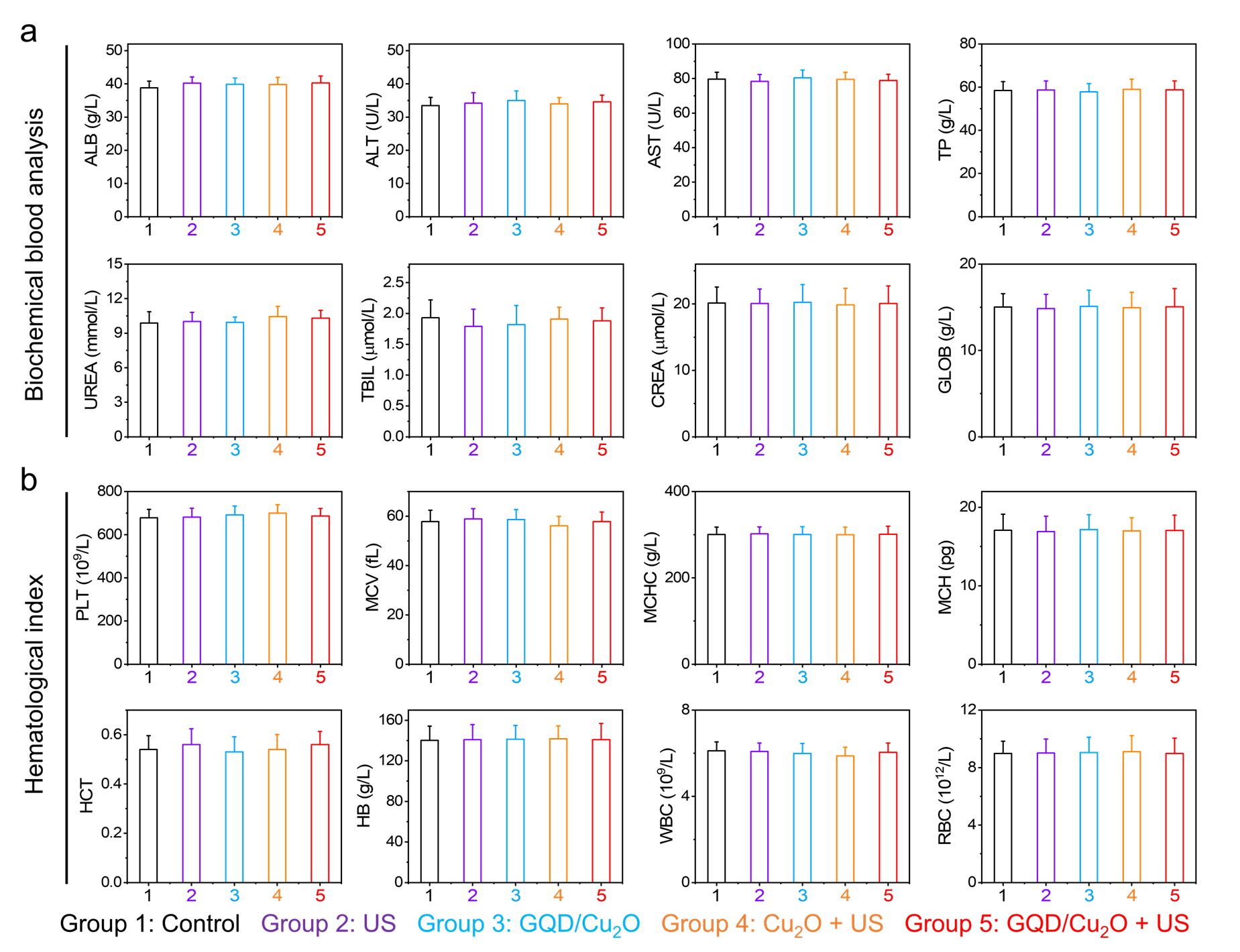


**Figure S37.** (a-b) Biochemical blood analysis (a) and hematological index (b) of the mice that were sacrificed at 18 days after different treatments (n=5 biologically independent samples). The terms of biochemical blood analysis include ALB, ALT, AST, TP, UREA, TBIL, CREA, and GLOB. The terms of hematological index include PLT, MCV, MCHC, MCH, HCT, Hb, WBC, and RBC. Data are presented as the mean ± SD. (n = 5).


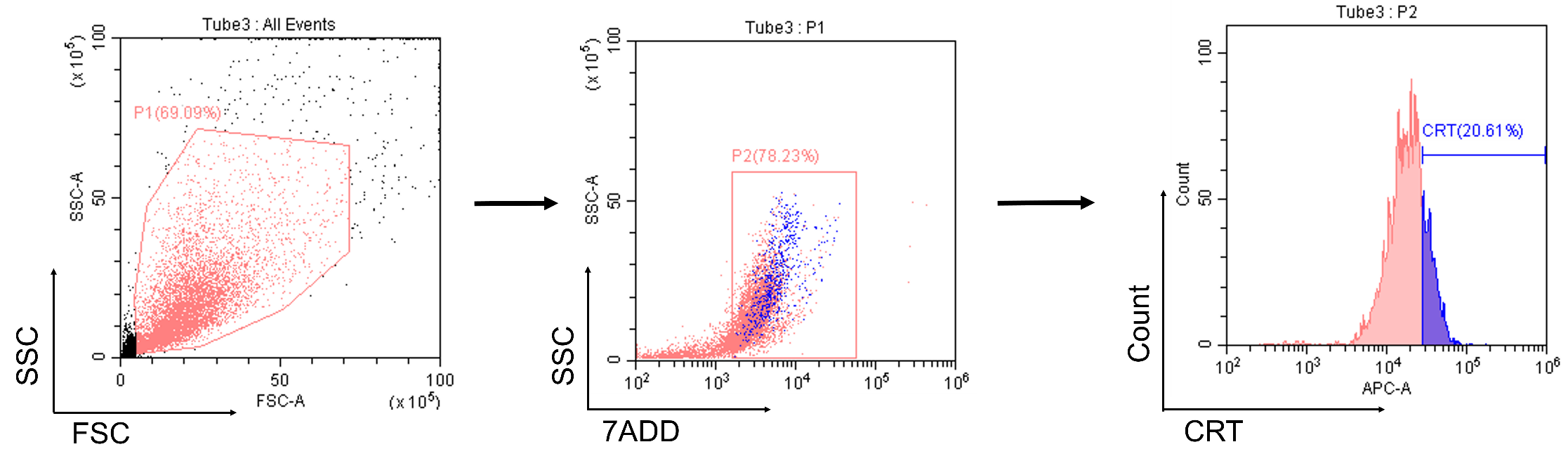


**Figure S38.** Gating strategy of flow cytometry analysis for determining CRT exposure level in 4T1 cells after different treatments.


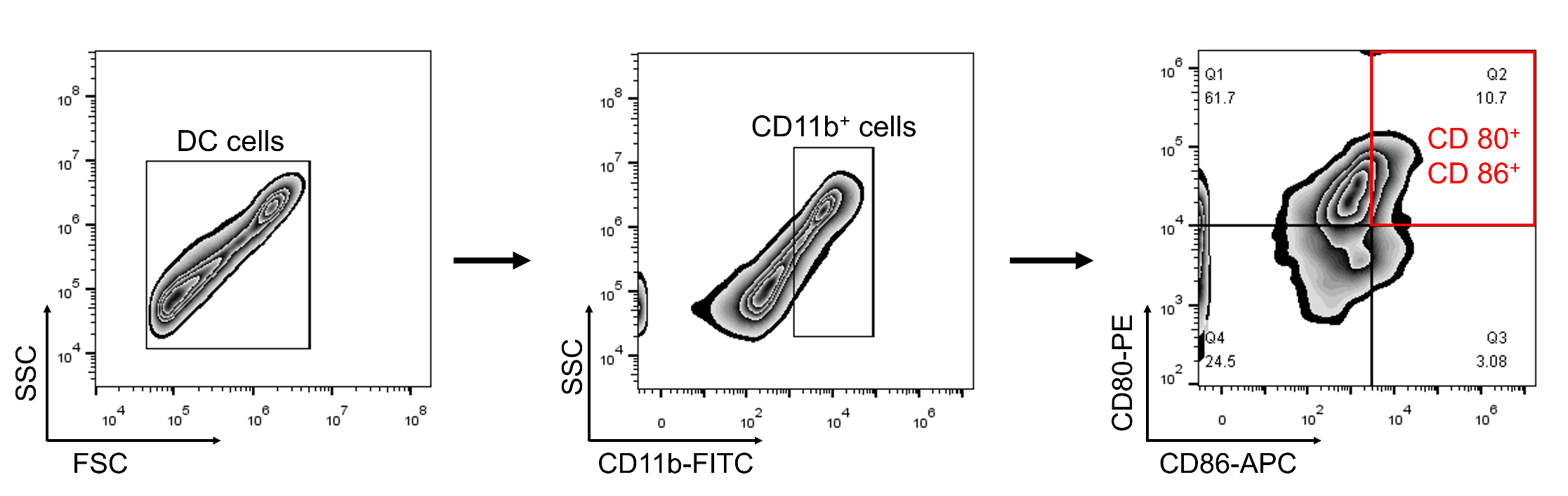


**Figure S39.** Gating strategy of flow cytometry analysis for determining the DC maturation.


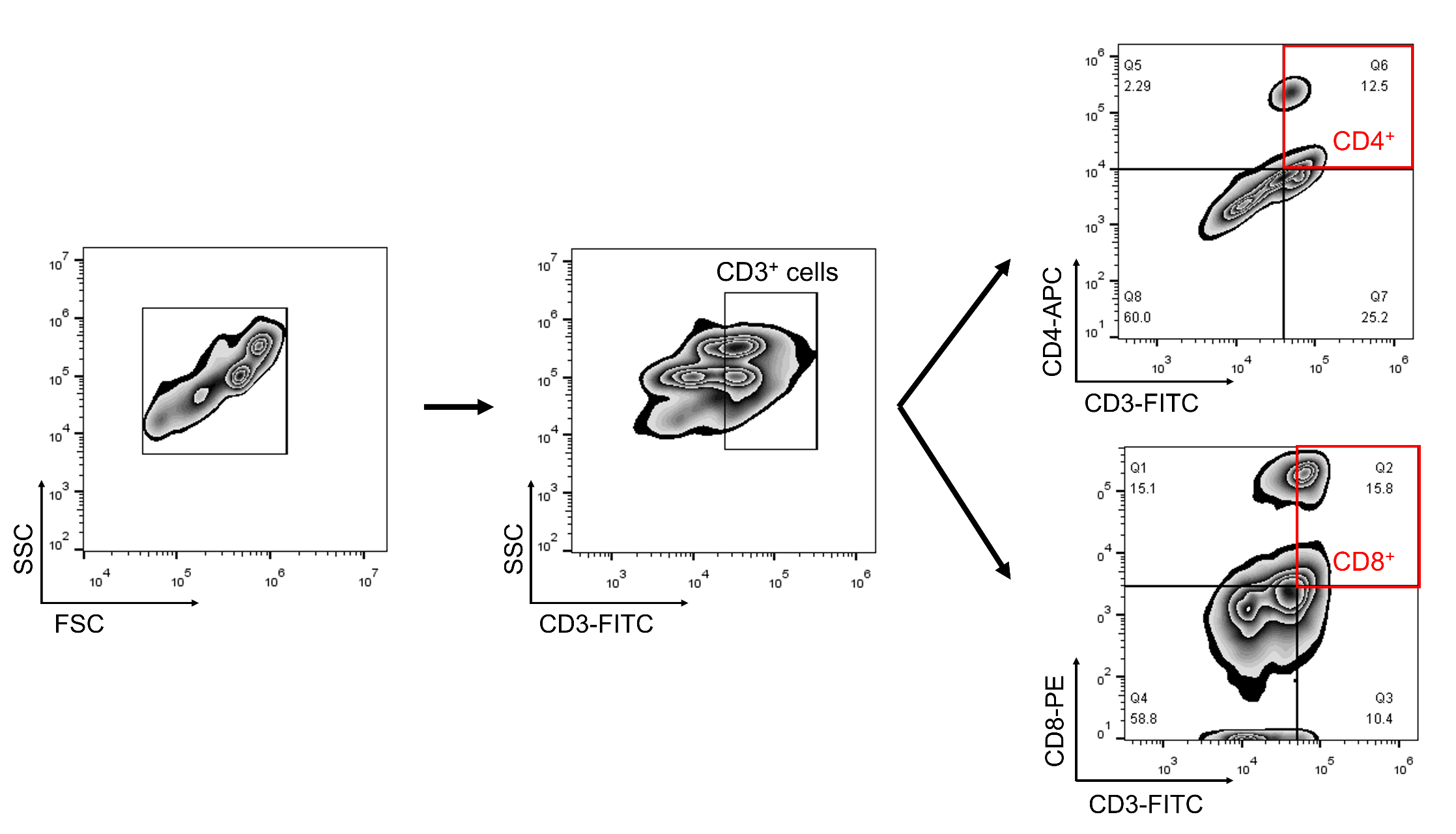


**Figure S40.** Gating strategy of flow cytometry analysis for determining the activation of T cells.
